# Purification, characterization, and preliminary serial crystallography diffraction advances structure determination of full-length human particulate guanylyl cyclase A receptor

**DOI:** 10.1038/s41598-022-15798-z

**Published:** 2022-07-12

**Authors:** Shangji Zhang, Debra T. Hansen, Jose M. Martin-Garcia, James D. Zook, Shuchong Pan, Felicia M. Craciunescu, John C. Burnett, Petra Fromme

**Affiliations:** 1 Center for Applied Structural Discovery, Biodesign Institute, Arizona State University, Tempe, AZ USA; 2 Center for Innovations in Medicine, Biodesign Institute, Arizona State University, Tempe, AZ USA; 3 Department of Crystallography and Structural Biology, Institute of Physical-Chemistry “Rocasolano”, Spanish National Research Council (CSIC), Madrid, Spain; 4 Cardiorenal Research Laboratory, Department of Cardiovascular Medicine, Mayo Clinic, Rochester, MN USA; 5 Department of Physiology and Bioengineering, Mayo Clinic, Rochester, MN USA; 6 School of Molecular Sciences, Arizona State University, Tempe, AZ USA

**Keywords:** X-ray crystallography, Structural biology, Membrane proteins

## Abstract

Particulate Guanylyl Cyclase Receptor A (pGC-A) is a natriuretic peptide membrane receptor, playing a vital role in controlling cardiovascular, renal, and endocrine functions. The extracellular domain interacts with natriuretic peptides and triggers the intracellular guanylyl cyclase domain to convert GTP to cGMP. To effectively develop methods to regulate pGC-A, structural information on the full-length form is needed. However, structural data on the transmembrane and intracellular domains are lacking. This work presents expression and optimization using baculovirus, along with the first purification of functional full-length human pGC-A. In vitro assays revealed the pGC-A tetramer was functional in detergent micelle solution. Based on our purification results and previous findings that dimer formation is required for functionality, we propose a tetramer complex model with two functional subunits. Previous research suggested pGC-A signal transduction is an ATP-dependent, two-step mechanism. Our results show the binding ligand also moderately activates pGC-A, and ATP is not crucial for activation of guanylyl cyclase. Furthermore, crystallization of full-length pGC-A was achieved, toward determination of its structure. Needle-shaped crystals with 3 Å diffraction were observed by serial crystallography. This work paves the road for determination of the full-length pGC-A structure and provides new information on the signal transduction mechanism.

## Introduction

Cardiovascular and metabolic diseases are the top-ranking diseases worldwide that are threatening peoples’ health and burdening their financial circumstances^1^. Approximately 10% of hypertensive patients are sub-classified as having resistant hypertension (RH), which is an uncontrolled hypertension state with high risk for heart failure, stroke, and metabolic diseases. To date, there are no FDA approved drugs for RH^2,3^. Further, there remains an unmet need for innovative new anti-hypertensive drugs with novel molecular mechanisms of action. Additionally, heart failure (HF) is the final outcome for most types of cardiovascular diseases. In the United States, total medical care costs for HF are estimated to increase from 31 billion US dollars in 2012 to 70 billion US dollars in 2030^4^.

The natriuretic peptides, which are natural hormones that are produced in the heart and are highly conserved across mammals, have been discovered for their main functions to control renal, metabolic and cardiovascular homeostasis^5–7^. Currently, three subtypes of natriuretic peptide receptors in mammals have been identified. They are natriuretic peptide receptor A (NPRA), natriuretic peptide receptor B (NPRB) and natriuretic peptide receptor C (NPRC). All three natriuretic peptide receptors are widespread in various tissues in response to both local and systemic physiological actions^8^. The natriuretic peptide hormones are structurally homologous and bind to specific natriuretic peptide receptors. Atrial natriuretic peptide (ANP) and B-type natriuretic peptide (BNP) bind with NPRA; C-type natriuretic peptide (CNP) binds with NPRB; and all three peptides bind with NPRC^9^.

NPRA and NPRB, also called particulate guanylyl cyclase A receptor (pGC-A) and B (pGC-B), respectively, are in the guanylyl cyclase family. pGC-A and pGC-B catalyze their intracellular guanylyl cyclase domains to convert GTPs into cellular messenger cyclic GMPs (cGMPs) upon binding with their natriuretic peptides^8,10^. In contrast, NPRC actively clears endogenous circulating natriuretic peptides via hydrolysis. Increased intracellular cGMPs stimulate and activate three cGMP-dependent effector molecules: cGMP-dependent protein kinases, cGMP-dependent ion-gated channels and cGMP-dependent phosphodiesterases. These cGMP-dependent signaling pathways may trigger different physiological responses such as sodium/water excretion, vasodilatation, anti-proliferation, anti-hypertrophy and anti-inflammation^11–15^. Additional actions involve metabolic regulation such as induction of lipolysis and browning of white adipocytes. Both ANP and BNP are the important hormones in activating pGC-A in body fluid, blood pressure and cardiac homeostasis^13,16,17^. The ANP/pGC-A signaling pathway lowers blood volume and blood pressure by increasing sodium/water excretion, vasorelaxation of vascular smooth muscle cells, increasing endothelial permeability and suppressing aldosterone^13,15,16^. Indeed, these actions of ANP have resulted recently in the engineering of a novel ANP analogue, which has recently been tested in human hypertension, underscoring the therapeutic opportunity of the ANP/pGC/cGMP pathway^18,19^.

To effectively develop novel pharmacological agents to regulate pGC-A, structural information is needed on its full-length form. The pGC-A monomer contains a peptide binding extracellular domain (ECD), a single helix transmembrane domain and an intracellular domain (ICD), which contains one protein kinase-like domain (PKLD) and a guanylyl cyclase catalytic domain (GCD)^20^. The current structural knowledge of pGC-A was made possible by earlier studies such as the milestone advance of Pandey and Kanungo (1993)^21^, who purified the mouse ECD and demonstrated its binding to ANP. Currently, the structure of only the extracellular domain (residues 1–435, out of 1057) of rat pGC-A is solved^22–24^. The relevant Protein Data Bank accession codes are 1DP4^22^ for *apo* rat pGC-A ECD; and 1T34^23^ and 7BRG-7BRJ^24^ for rat pGC-A ECD with various natriuretic peptide ligands. Based on the crystal structure information of rat pGC-A ECD with and without rat ANP, a homodimer rotation activation mechanism was proposed for full-length pGC-A, and it was suggested that full-length pGC-A also exists as a homodimer^23,25,26^. In comparison, data from two earlier references suggest a tetramer. the partially purified full-length pGC-A from bovine adrenal cortex tissue indicates that pGC-A exists as a disulfide-linked tetramer in its native state^27^. Such a higher oligomer structure may improve the local concentration of the receptor, effectively increasing ligand-binding sensitivity^27^. Additionally, crosslinking with disuccinimidyl suberate of human full-length pGC-A (expressed in the human HEK293 cell line) revealed a possible tetrameric complex in an immunoblot detection experiment^28^. One question that needs to be resolved is which oligomeric state is the native state of full-length pGC-A^29^.

With the detailed crystal structure information of the pGC-A ECD but lacking the structure information of the ICD, the actual signal transduction and activation mechanism of the ANP/pGC-A/cGMP pathway are still unclear. The knowledge of the full-length structure of the pGC-A receptor with and without the bound ANP is required to reveal the molecular basis of the function of the ANP/pGC-A/cGMP pathway. Importantly, pGC-A contains a guanylyl cyclase catalytic domain and is also classified in the membrane-bound guanylyl cyclase family of a super-family of single-span transmembrane receptors^6,26^. Many single-span transmembrane receptors use a ligand-induced association activation mechanism, in which the ligand triggers the receptor to bring the enzyme close to active site. The rotation mechanism initiating pGC-A signaling transduction and activating the guanylyl cyclase catalytic domain by rotation signaling is the first mechanism identified within single-span transmembrane receptors^25,26^. Also, the membrane-bound guanylyl cyclase structure information is yet to be determined.

In seeking to solve the structure of the human full-length pGC-A and resolve the ANP/pGC-A/cGMP signal transduction mechanism, we established the full expression and purification protocol for the full-length human pGC-A receptor. We also successfully screened the crystallization conditions and report here the first X-ray diffraction of crystals of human full-length pGC-A. In addition to characterizing the oligomeric state of full-length pGC-A, the first in vitro cGMP functional assay results revealed that the purified tetramer in the detergent micelle solution was functionally active. Furthermore, the functional assay also suggested that the binding ligand moderately activates pGC-A, and that ATP is not crucial for the activation of guanylyl cyclase.

## Results

### Expression of pGC-A was designed and optimized

To express and purify functional human pGC-A for structural purposes, Sf9 insect cells were chosen as the protein expression system. The detailed construct design and expression clone details for the full-length pGC-A are in Supplementary Figs. S1A,B and S2A,B. In this expression construct, the predicted signal peptide (amino acid residues 1–32) was replaced with the highly expressing hemagglutinin signal peptide (MKTIIALSYIFCLVFA), a decahistidine purification tag (HHHHHHHHHH), a short linker of three residues (GAP, encoded by an *Asc*I cloning site), and a tobacco etch virus (TEV) protease cleavage site (ENLYFQG). The remaining protein sequence contains residues 33–1061 of pGC-A, including the transmembrane domain at residues 474–495. Generated recombinant bacmid DNA and bacmid PCR size confirmation are shown in Supplementary Fig. S3.

### Optimization of pGC-A expression levels

**Figure 1:**
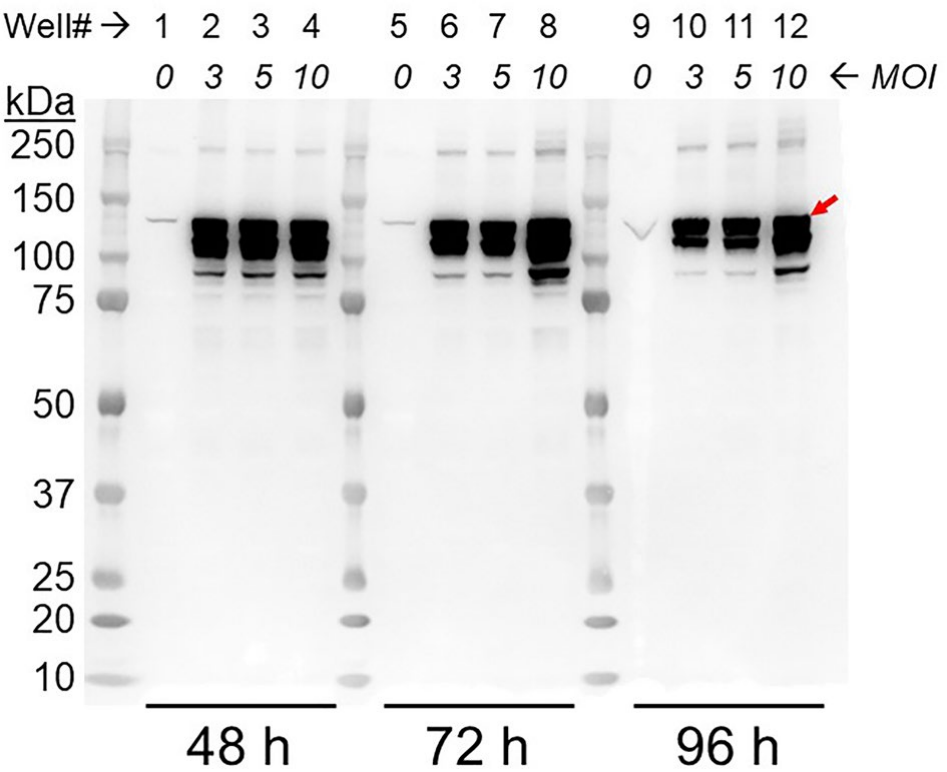
Optimization of pGC-A expression in Sf9 cells. Western blot (anti-pGC-A) of total cell protein over different virus multiplicities of infection (MOI) and transfection time. MOI of 0 indicates no virus transfection. pGC-A (red arrow) is approximately 120 kDa.

Sf9 insect cell expression of pGC-A was tested in small-scale expressions as follows. The different multiplicity of infection (MOI) of 0, 3, 5, 10 was used to transfect 4 mL of insect cell culture. Transfected cells were collected at 48, 72, and 96 h. A western blot of total cell protein from these samples showed that the best expression parameter combination was MOI of 10 and harvest at 72 h (Fig. 1, lane 8). The MOI of 3 and 5 showed similar protein expression levels within same post-transfection time (Fig. 1, compare lanes 2 with 3; 6 with 7; and 10 with 11), with slightly larger yields at an MOI of 10 for transfection times 72 h and 96 h (lanes 8 and 12, respectively), but not for 48 h (lane 4). The pGC-A expression level generally decreased as the post transfection hours increased (Fig. 1, compare lanes 2–4 with 6–8 and with 10–12). This is probably due to the self-lysis of transfected cells during virus transfection. The longer the viruses were transfected in cells, the higher the likelihood of cell lysis. The different sized bands between 150 and 100 kDa may be caused by inconsistent glycosylation levels and protease activity. These results show that pGC-A was successfully expressed in Sf9 cells at all MOI’s and from 48 to 96 h after transfection.

### Recombinantly expressed pGC-A is functional in a whole cell activity assay

**Figure 2:**
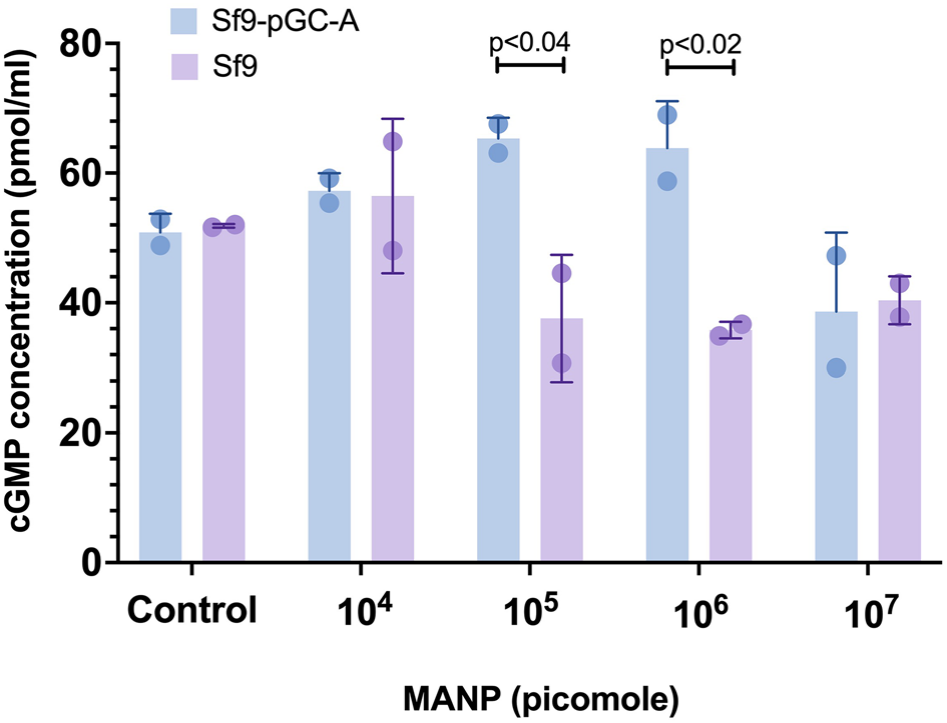
Determination of expressed full-length pGC-A functionality via whole-cell activity assay. The competitive ELISA assay measured the cGMP yield level in Sf9 cells expressing full-length pGC-A versus control Sf9 cells (n = 2). Both cell types were incubated with different concentrations of MANP ligand (0 to 10^7^ pmol). Incubation with 0 pmol MANP served as a negative control.

To test if expressed full-length pGC-A is functional in Sf9 cells, the whole-cell functional activity assay was performed based on measuring the produced cGMP concentration via a competitive ELISA assay (Fig. 2). Transfected cells expressing pGC-A (Sf9-pGC-A), or non-transfected cells (Sf9), were incubated with different concentrations of MANPs (mutant ANP), which is a frameshift mutant natriuretic peptide based on the cardiac hormone ANP^30^. MANP contains a C-terminal extension that increases binding to the receptor and causes decreased binding to its counterplayer neprilysin, which degrades ANP^30^. In Fig. 2, cells that expressed active pGC-A generated a higher cGMP level when compared with non-transfected Sf9 cells. There was a statistically significant difference between cells expressing full-length pGC-A and non-transfected cells when cells were incubated with 10^5^ and 10^6^ pmol MANP (Fig. 2). However, on increasing the MANP concentration to 10^7^ pmol, the level of cGMP in both cells was lower than in the control group (basal cGMP level). This may be caused by toxicity induced by high-dose MANP, which could become toxic to the cells. In conclusion, the whole-cell activity assay showed that the expressed full-length pGC-A was functionally active in the Sf9 cell membrane.

### Large-scale purification of full-length pGC-A

**Figure 3:**
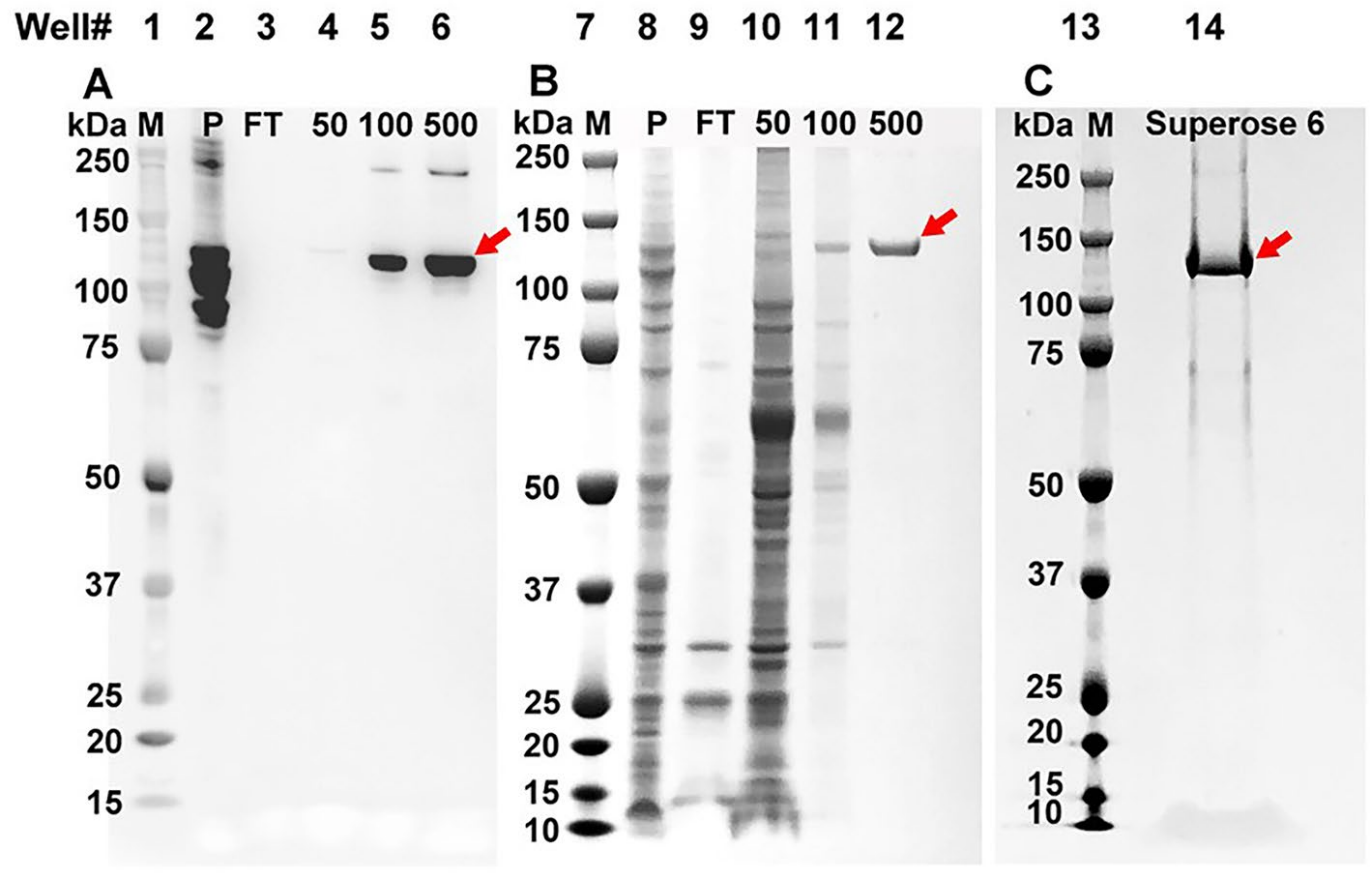
Purification of full-length pGC-A via affinity and size exclusion columns. (**A**) Western blot (anti-pGC-A) of samples from cell lysis to the affinity column. pGC-A (red arrow) is ~ 120 kDa. (**B**) Coomassie blue stain from cell lysis to the affinity column. (**C**) Coomassie blue stain of the eluted fraction from Superose 6 that was used for protein crystallization. M: marker; P: membrane pellet from ultracentrifugation after solubilization with n-dodecyl-β-D-maltoside (DDM) and cholesteryl hemisuccinate (CHS); FT: flowthrough from the affinity column; 50, 100, 500: imidazole (mM) at elution from the affinity column.

To purify full-length pGC-A, a culture with 1.5 L of 2 × 10^6^ cells/mL of transfected Sf9 insect cells was prepared and cells were harvested 72 h post-infection. Harvested cells were lysed for cell plasma membrane isolation. The isolated membrane was solubilized with detergent yielding the pGC-A receptor in the form of protein-detergent micelles. Solubilized full-length pGC-A was purified by nickel-affinity chromatography and size exclusion chromatography using a Superose 6 column. After membrane solubilization, samples from each step of the purification procedure were collected and analyzed by gel electrophoresis. Western immunoblotting results are presented in Fig. 3A, and Coomassie-stained SDS-PAGE results are presented in Fig. 3B,C.

The western blotting (anti-pGC-A) results revealed that large quantities of full-length pGC-A were still present in the membrane pellets (Fig. 3, lane 2). The detergent-solubilized membrane supernatant underwent gravity nickel affinity chromatography (Fig. 3, lanes 3–6). No pGC-A was visible in the column flowthrough (Fig. 3, lanes 3 and 9). These results indicated that solubilized pGC-A was well-captured in the affinity column. After washing the affinity column with purification buffers containing 50 and 100 mM imidazole (Fig. 3, lanes 4, 5, 10 and 11), pGC-A was eluted with the purification buffer containing 500 mM imidazole (Fig. 3, lanes 6 and 12). The Coomassie blue-stained results showed that the majority of contaminant proteins were washed off with the purification buffer containing 50 mM imidazole (Fig. 3, lane 10). Although some pGC-A was present in the 100 mM imidazole wash (Fig. 3, lane 5), this wash also contained many other contaminants (Fig. 3, lane 11). Sample eluted from the buffer containing 500 mM imidazole showed a single protein band in the Coomassie blue-stained gel (Fig. 3, lane 12). The western blotting results indicated that the single band represented the full-length pGC-A protein (Fig. 3, lane 6). This purified pGC-A was concentrated and further purified using the Superose 6 size exclusion column. The peak fractions from Superose 6 columns were combined and are shown in Fig. 3, lane 14. Altogether, the full-length pGC-A was successfully purified.

### Crystallization optimization

**Figure 4:**
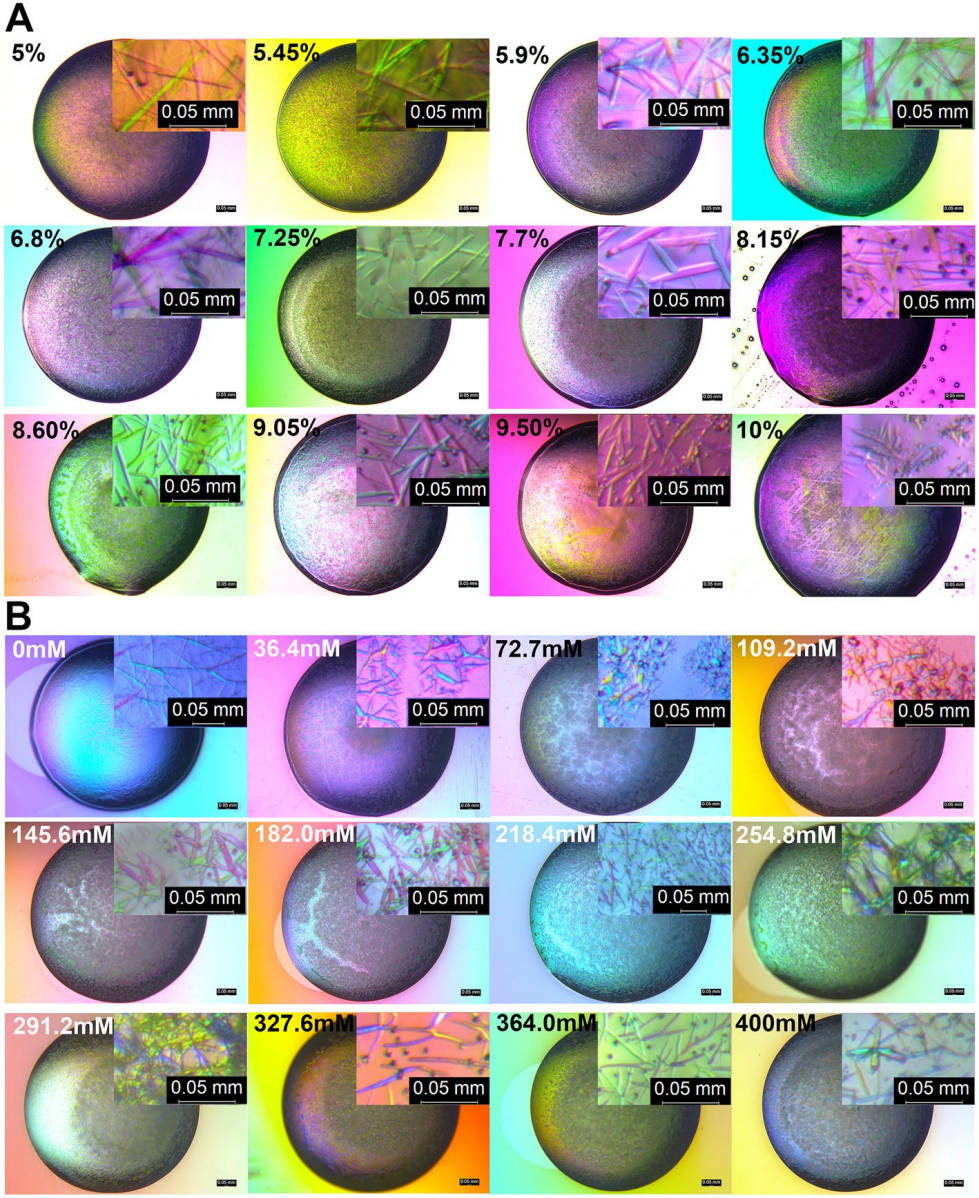
Summary of both PEG 3350 and ammonium sulfate narrow crystallization screening derived from the initial crystallization hits. Shown are visible light images of crystallization drops in 24-well plates using a protein concentration of 1.25 mg/mL. (**A**) Screening of 5–10% PEG 3350. (**B**) Screening of 0–0.4 M ammonium sulfate.

To further optimize the best initial crystallization hits, which were observed in 0.32 M ammonium sulfate, 0.08 M of MES buffer, pH 6.5, 8% PEG 3350 that was identified in a broad screen at 1.18 mg/mL and 2.25 mg/mL protein (Supplementary Figs. S4A,B and S5A,B), narrow gradient screenings were applied to further optimize the PEG 3350 and ammonium sulfate concentrations at a lower protein concentration, 1.25 mg/mL. The narrow PEG 3350 screening was set from 5 to 10% (Fig. 4A), and the narrow ammonium sulfate screening was set from 0 M to 0.4 M (Fig. 4B). In the narrow PEG 3350 screening, the PEG range of 5%–9.05% grew better needle-shaped crystals (Fig. 4A). A trend where higher PEG concentration induced smaller needle-shaped crystals to form was noted (Fig. 4A). The narrow ammonium sulfate screening indicated that ammonium sulfate salt was necessary to form needle-shaped crystals, since 0 M salt yielded no crystals (Fig. 4B). There were many clusters of needle-shaped crystals formed at lower ammonium sulfate concentrations (Fig. 4B). It can be postulated that ammonium sulfate facilitated protein crystal formation in the drops after phase separation occurred in the crystallization drops (Fig. 4B, 36.4 mM to 145.6 mM). The ammonium sulfate concentrations from 218 to 291 mM produced better crystals in the drop without forming clusters or precipitation zones in the center of the drops. Comparing the higher protein concentration (Supplementary Fig. S5B) with the lower one (Fig. 4B), the center precipitation zones were much smaller with the lower protein concentration condition and the high ammonium sulfate concentrations. In conclusion, to crystalize the full-length pGC-A with a lower protein concentration, the optimal PEG 3350 concentration ranged from 5 to 10%, and the optimal ammonium sulfate concentration ranged from 218 to 400 mM.

### Confirmation that pGC-A protein formed crystals

**Figure 5:**
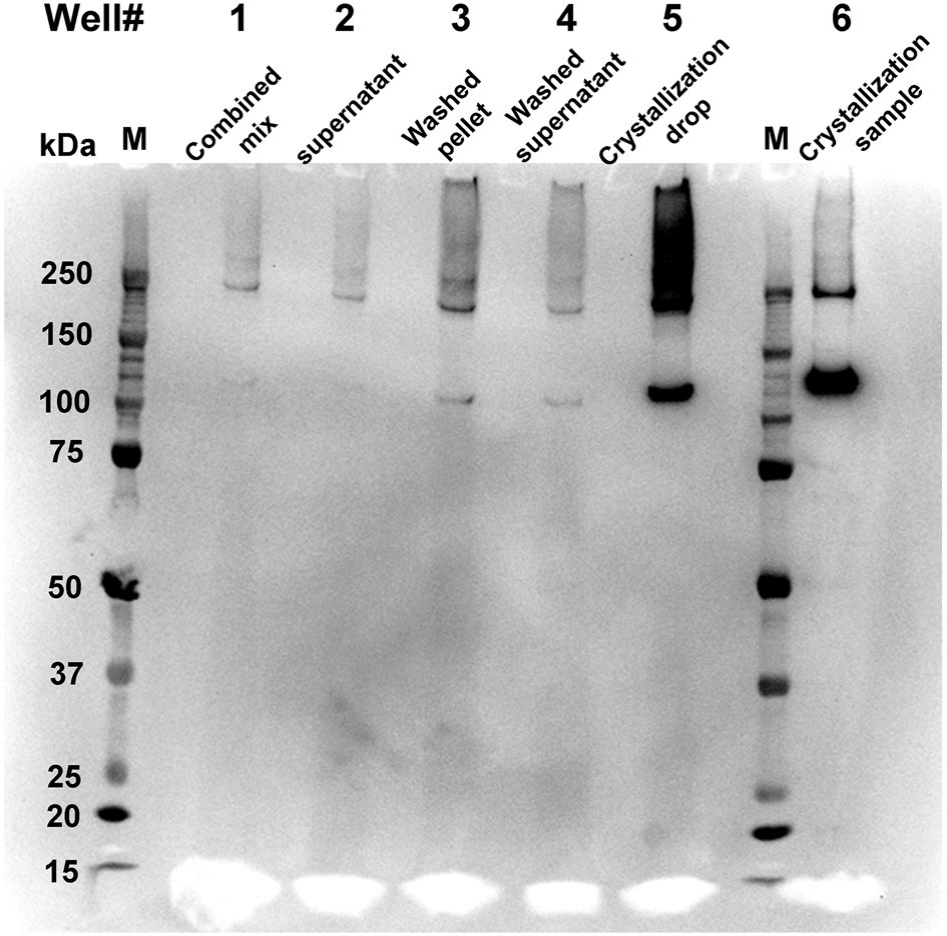
Presence of pGC-A in crystals was confirmed via western blot. The anti-pGC-A antibody was used in the western blot. The pGC-A monomer is 120 kDa. Lane 1: combined mix: combined crystallization drops collected in the PCR tube. Lane 2: supernatant: the supernatant collected from the centrifuged combined mix. Lane 3: washed pellet: the crystal pellet was washed with the precipitant solution and collected again via centrifugation. Lane 4: washed supernatant: the supernatant from washed crystal pellet. Lane 5: crystallization drop: one crystallization hanging drop directly mixed with SDS sample buffer. Lane 6: crystallization sample: purified pGC-A before crystallization, serving as a positive control.

To confirm the needle-shaped crystals formed under the screening condition were composed of purified full-length pGC-A protein, the crystals were isolated from combined crystallization drops via centrifugation. The combined crystal pellet was washed with the precipitant solution and pelleted down again for western blot signal determination. During the crystal collection and washing process, the sample from each step was mixed with sodium dodecyl sulfate (SDS) sample buffer to detect the pGC-A protein via western blotting. Figure 5 shows the anti-pGC-A western blot results and confirms that the protein crystals were formed by pGC-A. In Fig. 5, lanes 1, 2, and 4 had weaker western blot signals than Fig. 5, lane 3, representing the pelleted crystals. The weaker pGC-A signal was caused by fewer pGC-A proteins in the sample. In Fig. 5, lane 3 contained the strongest pGC-A signal during the crystal collection and washing process compared to lane 1 (the combined crystallization drops), lane 2 (the supernatant collected from the centrifuged combined mix), and lane 4 (the supernatant from washed crystal pellet), which indicated that most of the pGC-A was in the pelleted crystals (Fig. 5, lane 3). The weak western blot signals in the supernatant samples (Fig. 5, lanes 2 and 4) were likely from the un-crystallized pGC-A protein in solution, and shear force partially dissolved needle-shaped crystals that resulted from pipetting. Unlike salt crystals, protein crystals are fragile and tend to break from pressure^31^. The weak western blot signals in Fig. 5, lane 4, were probably caused by resuspending the crystals with the precipitant solution by pipetting. Many microcrystals were stuck on the cover glass during the crystal drop collection/harvesting process, making transfer into the PCR tube a challenge.

To test the pGC-A signal from the entire crystallization drop, SDS sample buffer was directly added to one crystallization drop (Fig. 5, lane 5). The directly mixed crystallization drop (Fig. 5, lane 5) had a much higher pGC-A western blot signal than the washed crystal pellet (Fig. 5, lane 4). This was caused by formed crystals sticking on the hanging drop silicon coverslip, which were difficult to transfer into the PCR tube. In Fig. 5, lane 6 contains purified pGC-A protein before crystallization and served as a positive control. The supernatant (Fig. 5, lane 2) would have a strong signal similar to the crystallization drops (Fig. 5, lane 5) if pGC-A protein did not form crystals and stayed in solution. Additionally, in Fig. 5, lanes 3 and 5 showed strong pGC-A signals above the 250 kDa molecular weight in the western blot. Nevertheless, the main protein signal band in the sample of the purified protein before the crystallization (Fig. 5, lane 6) was 120 kDa, consistent with the monomer size of full-length pGC-A. The higher molecular weight bands represented higher oligomeric states of pGC-A. Indeed, natural dimerization of pGC-A was observed during pGC-A purification (Fig. 3) and is also seen in the positive control (Fig. 5, lane 6). Interestingly, in Fig. 5, both lanes 3 and 5 showed much higher oligomeric states in the crystal samples. Possibly, these higher molecular weight signals may represent the higher oligomeric states that dominated in the protein crystals.

Notably, samples loaded on the SDS-PAGE were incubated in SDS without the heating process. The native protein oligomeric state may be stabilized against SDS denaturation in the crystals. Additionally, the crystallization plate was set up three weeks prior and stored in the cold room. Full-length pGC-A is easily degraded in solution, but there was no pGC-A degradation signal (western blot signal below 120 kDa) detected in the western blot from the crystals (Fig. 5). Collectively, the western blot results indicated that pGC-A formed needle-shaped crystals, and that the protein is stabilized in its native oligomeric form in the crystals.

### Diffraction patterns of pGC-A crystals via serial crystallography

**Figure 6:**
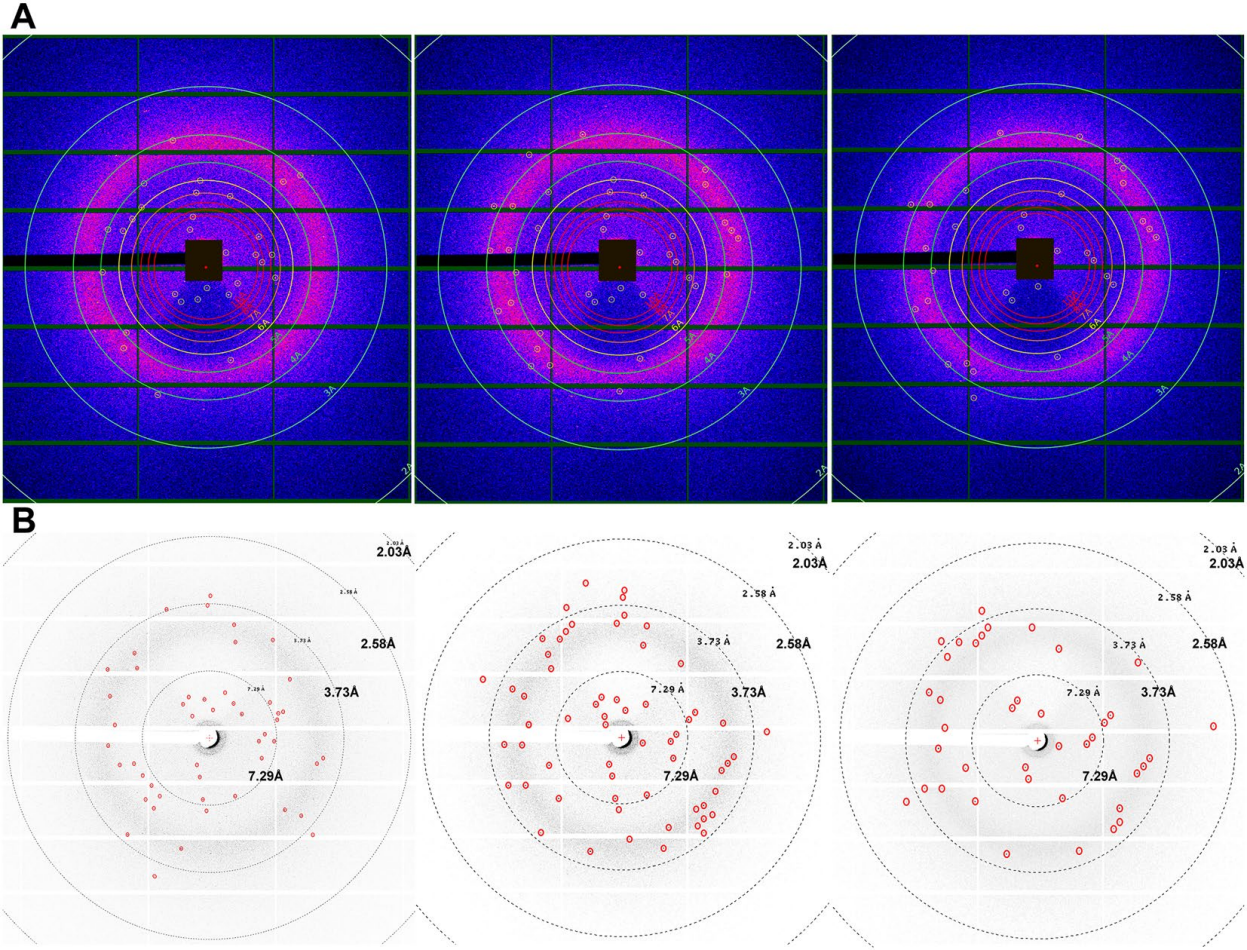
Indexed diffraction patterns from pGC-A microcrystals collected via serial crystallography at the Advanced Photon Source. Three indexable diffraction patterns with the highest resolution of 3 Å. (**A**) Blue/pink diffraction graphs show diffraction patterns analyzed using CrystFEL software. (**B**) The original diffraction patterns shown with white background. All diffraction dots were manually circled in red for better visualization.

Harvested needle-shaped crystals grown under the optimized conditions were shipped to the Advanced Photon Source (APS) at Argonne National Laboratory in a temperature-controlled container. Unlike other membrane proteins directly crystallized in the lipidic cubic phase (LCP), harvested pGC-A crystals must be embedded into LCP before delivering to the X-ray source. The final delivery LCP was prepared by mixing the crystal solution (40%) with molten monoolein lipid (60%) in a glass dual-syringe lipid mixer until a homogeneous transparent LCP was formed. A specialized LCP injector was used to deliver the LCP-crystal mixture in a serial crystallography experiment^32,33^. During the serial crystallography experiment at APS, LCP-embedded crystals are constantly jetted perpendicular to the X-ray beam. Unlike traditional single-crystal diffraction, in the serial crystallography method, single crystal snapshots in random orientation are collected from a stream of microcrystals, and data sets consist of diffraction patters from thousands of individual microcrystals. Each diffraction pattern is thereby obtained from a different crystal. During this specific experiment, LCP-embedded pGC-A crystals were jetted across the X-ray source for approximately 2 h. A total of 270,000 frames were recorded on the detector. Five diffraction patterns were detected for the first time for full-length pGC-A crystals. Accordingly, three out of five recorded diffraction patterns could be indexed by the CrystFEL software^34^ (Fig. 6A). Based on the three indexable diffraction patterns, the CrystFEL indexing results showed that the needle-shaped crystals likely represent crystals in the P1 space group. The highest diffraction spot reached 3 Å (Fig. 6B, rightmost image).

### Different pGC-A oligomeric states were observed in size exclusion chromatography

**Figure 7:**
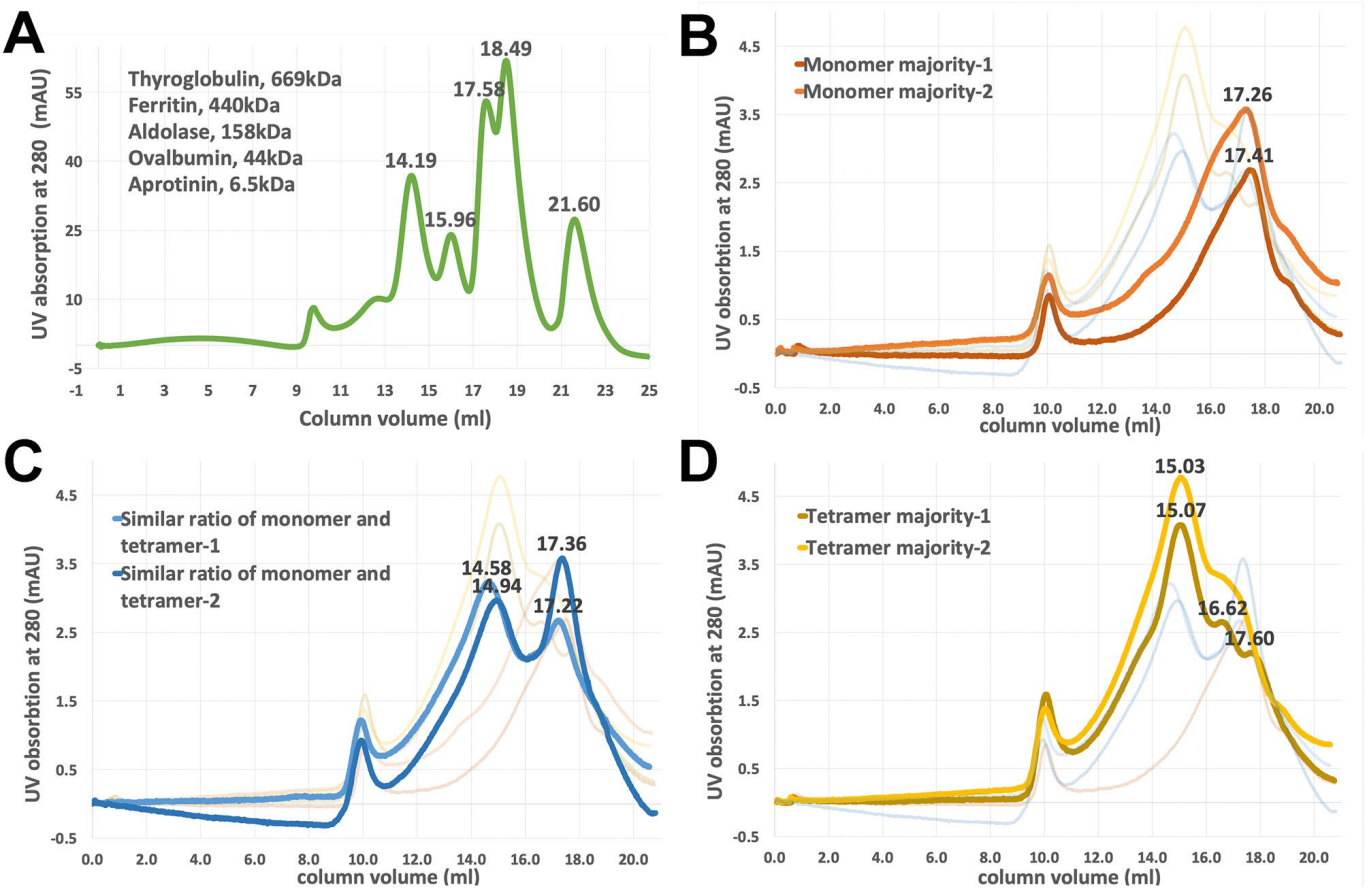
Dynamic oligomeric states of pGC-A seen in replicate runs of Superose 6 size exclusion chromatography may be dependent on protein concentration. (**A**) The Superose 6 10/300GL column performance profile. Five standard proteins were used to generate relative molecule elution points based on different molecular sizes. Thyroglobulin (669 kDa) eluted at 14.19 mL, ferritin (440 kDa) eluted at 15.96 mL, aldolase (158 kDa) eluted at 17.58 mL, ovalbumin (44 kDa) eluted at 18.49 mL, and aprotinin (6.5 kDa) eluted at 21.60 mL. (**B-D**) Size exclusion chromatography of pGC-A. (**B**) The peak intensity at 17.3 mL corresponds to the pGC-A monomeric state (120 kDa). pGC-A monomer is the major peak determined by chromatography. Other ratios were faded out in the background and served as supplemental comparison. (**C**) pGC-A tetramer and monomer present similar ratios in the chromatographic separation. The peak intensity at 14.76 mL and 17.29 mL corresponds to pGC-A tetrameric (480 kDa) and monomeric states, respectively. (**D**) The pGC-A tetramer is the major peak. The peak intensity at 15.05 mL corresponds to the pGC-A tetrameric state.

In each full-length pGC-A purification experiment, the Superose 6 size exclusion column was extensively used to purify pGC-A before crystallization. Interestingly, different oligomeric states were observed under the same purification protocol. To determine the relative molecular weight of the different oligomers, five molecular weight standard proteins, ranging from 6.5 kDa for aprotinin to 669 kDa for thyroglobulin, were injected into the Superose 6 column to generate the column performance elution profile (Fig. 7A). Upon comparing the Superose 6 column standard chromatography profile (Fig. 7A) with the pGC-A oligomer elution profiles (Fig. 7B–D), the results suggested that the pGC-A oligomers were either tetramer (480 kDa) or monomer (120 kDa).

The chromatography profiles in Fig. 7B–D summarize three common full-length pGC-A oligomeric states during the Superose 6 purification. In Fig. 7B, the major peak was eluted around 17.3 mL. The elution volume was remarkably close to the standard aldolase protein (158 kDa) elution profile at 17.58 mL, which indicated that the majority peak in the Fig. 7B represents the monomer size of full-length pGC-A. As shown in Fig. 7D, the majority peak was eluted around 15.0 mL, with a minor shoulder peak around 17.1 mL (monomer). The majority peak elution volume was extremely close to the standard ferritin protein (440 kDa) elution profile at 15.96 mL, indicating that the majority peak in Fig. 7D represents the tetramer size of full-length pGC-A. In Fig. 7C, two prominent elution volume peaks can be observed at approximately 14.76 mL and 17.29 mL, representing the tetramer and monomer size, respectively. In addition, a minor peak was eluted at 16.62 mL in Fig. 7B. It may represent the dimer state of full-length pGC-A. However, it is difficult to define the size due to the Superose 6 resolution limitation. In addition, the possible dimer peak was rarely observed during the replicate purifications. Most peaks represented either the tetramer or monomer.

Collectively, the Superose 6 elution chromatography profiles of full-length human pGC-A could be classified as the tetramer majority group (Fig. 7D), monomer majority group (Fig. 7B), and a separation that contains similar ratio of tetramer and monomer group (Fig. 7C). This unique and distinctive protein characteristic may be dependent on the protein concentration. On comparing the peak heights and UV absorption levels (y-axes) between Fig. 7B-D, it was observed that the higher the protein concentration in the injected pGC-A sample, the larger the size of the earlier eluting peak, which represents the tetramer. Similar results were also indirectly observed during injection of the concentrated elution sample of the affinity chromatography into the Superose 6 column. The smaller sample volume (approximately 300 μL) and more concentrated the protein was before injection, the larger the tetramer peak size observed; the larger the sample volume (approximately 450 μL), and the lower the protein concentration, the smaller the peak size was observed for the tetramer.

That pGC-A oligomerization was dependent on protein concentration was additionally supported by dynamic light scattering. From one Superose 6 separation that had three peaks (Supplementary Fig. S6A, peaks P2, P3 and P4) corresponding to the monomer, dimer and tetramer, pGC-A was present in each peak as shown by Western blot or Coomassie (Supplementary Fig. S6B). The protein in each peak was concentrated, as necessary for dynamic light scattering. Each concentrated peak demonstrated a uniform and straight pattern in the heat map and the same radius reading at 11 nm (Supplementary Fig. S6C). These results are consistent with each peak forming a higher oligomeric state during the concentration step, and further demonstrate monodispersity of the purified pGC-A, which is an important characteristic for successful crystallization.

Interestingly, this concentration-dependent protein oligomerization phenomenon has also been observed with the ECD of pGC-A. Several research groups have reported that purification of the ECD of pGC-A showed different oligomeric states during size exclusion chromatography^22,35,36^. The oligomerization was concentration-dependent, and the dimerization affinity can be further enhanced upon hormone binding^22,35,36^. In addition, self-oligomer formation has been found to occur during the purification of the ECD of natriuretic peptide receptor C^37^.

### Full-length pGC-A high oligomeric state confirmed as a tetramer

**Figure 8:**
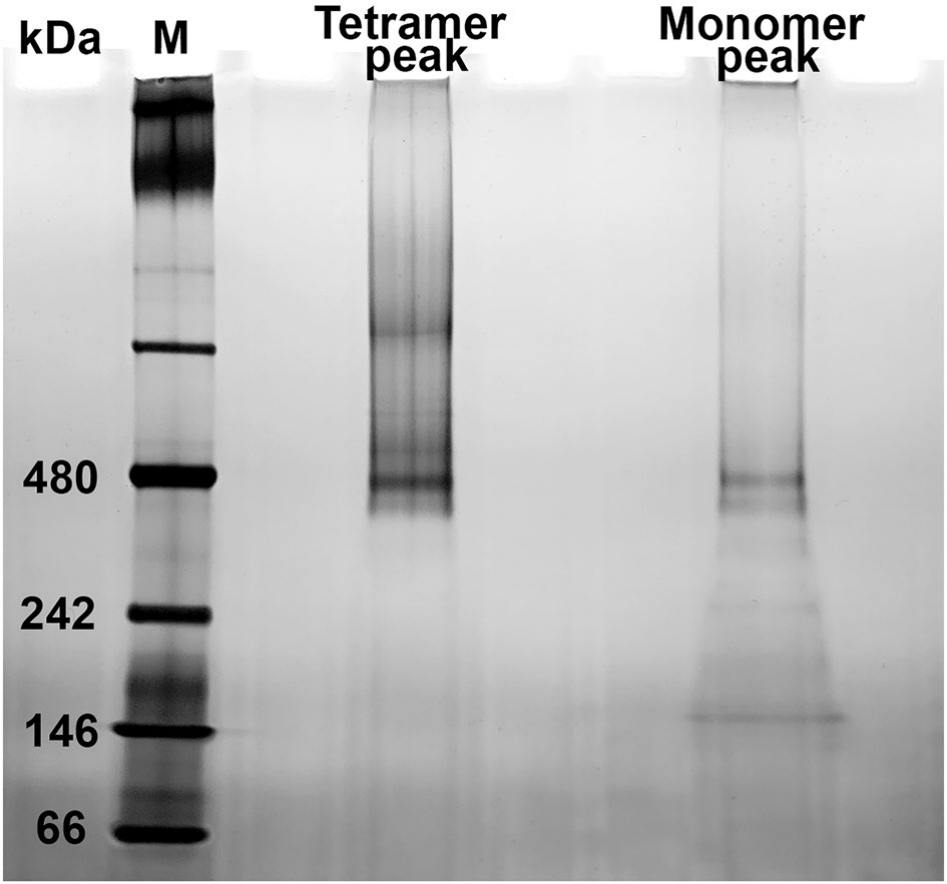
Silver stain of high resolution clear-native PAGE from pGC-A Superose 6 fractions. Superose 6 column eluted tetramer (480 kDa) and monomer (120 kDa) size peaks were concentrated and analyzed in a 4–16% native gel. M, marker.

To verify whether the full-length pGC-A high oligomer state eluted from the Superose 6 column represented the true tetramer and that the protein concentration truly converted the monomer into higher oligomers, two peak fractions (representing the tetramer size and monomer sizes) were eluted from Superose 6. These two elution fractions were collected and concentrated separately for high resolution clear native polyacrylamide gel electrophoresis (hrCN-PAGE) analysis. The prepared tetramer and monomer peak samples were loaded on a 4–16% clear native gel, followed by silver staining (Fig. 8). The silver-stained hrCN-PAGE results revealed that the tetramer peak fraction sample featured a strong signal band at 480 kDa, representing a tetramer (Fig. 8). Interestingly, the sample from the concentrated monomer size peak also showed a strong tetramer size band signal (Fig. 8). This result revealed that protein concentration converted the monomer into a higher oligomer state. In addition, no significant homodimer (240 kDa) band signal was observed in either the tetramer or monomer peak samples in Fig. 8. In conclusion, the higher oligomeric state peak sample from the Superose 6 chromatography was confirmed as a tetramer. Moreover, the protein concentration contributed to converting monomer into the higher oligomer (tetramer) state.

### Full-length pGC-A tetramer was not oligomerized through disulfide bonds

**Figure 9:**
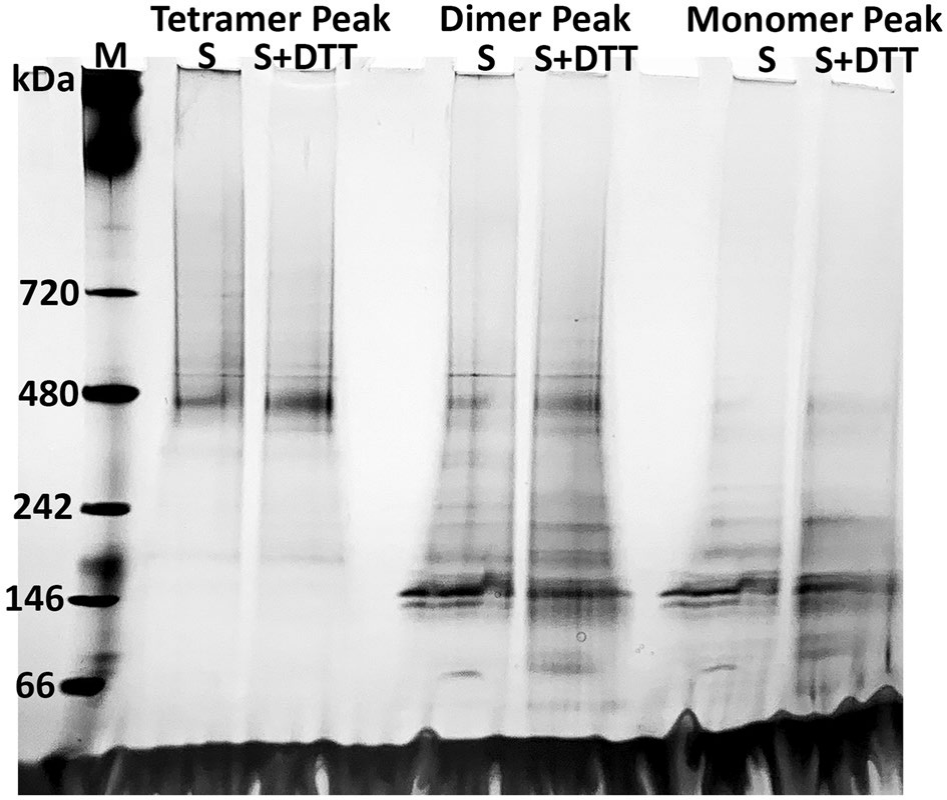
Silver stained clear-native PAGE of different oligomeric samples treated with or without dithiothreitol (DTT) overnight. Three peak samples, which represent the tetramer, dimer, and monomer of full-length pGC-A, were concentrated and split in half for treatment with or without DTT. S, concentrated sample only; S + DTT, concentrated sample incubated with 1 M DTT overnight.

In a previous study, the partially purified bifunctional pGC-A from bovine adrenal cortex tissue was proposed to exist as a disulfide-linked tetramer in its native state^27^. It is unclear if all cysteine residues are involved in the formation of intramolecular or intermolecular disulfide bonds. To test if the formation of intermolecular disulfide bonds caused the tetrameric oligomeric state, no reducing reagent was added in this specific purification experiment. Based on the Superose 6 column standard elution profile (Fig. 7A), three peak fractions from each peak, which represented the tetramer (480 kDa), dimer (240 kDa), and monomer (120 kDa) size, were collected separately. Each concentrated peak fraction was further split into two portions. One portion contained the sample only, and another portion was mixed with 1 M dithiothreitol (DTT). All samples were incubated on ice overnight then loaded on the native gel. The silver-stained hrCN-PAGE revealed no difference in all three peak samples, both with and without DTT (Fig. 9). Accordingly, the tetrameric oligomer is not caused by a disulfide bond. Furthermore, in the dimer size peak sample, a potent protein signal was observed in the form of tetramer and monomer size bands, with only a minor dimer peak (Fig. 9). These results indicated that the dimer is rarely formed when compared with tetramer and monomer. Collectively, these results showed that the pGC-A tetramer is not oligomerized through disulfide bonds. Furthermore, the rarely observed dimer in the Superose 6 column elution profile was not due to the column resolution limit. The results imply that the dimer is not a favored oligomeric state.

### Purified full-length pGC-A tetramer was functionally active in an in vitro activity assay

Previous studies have shown that ATP binds to the intracellular protein kinase-like domain (PKLD) and plays a regulatory role in the activation of the pGC-A guanylyl cyclase catalytic domain (GCD)^38–40^. Subsequently, more detailed studies of the regulation of pGC-A by ATP have proposed that the ATP dependent ANP/pGC-A/cGMP signal transduction mechanism may be a two-step ordered process^41^. First, activation of the ATP-dependent allosteric step partially activates the GCD through binding of ATP to the ATP regulatory module domain; second, the phosphorylation of the cyclase fully activates the guanylyl cyclase^41,42^.

**Figure 10:**
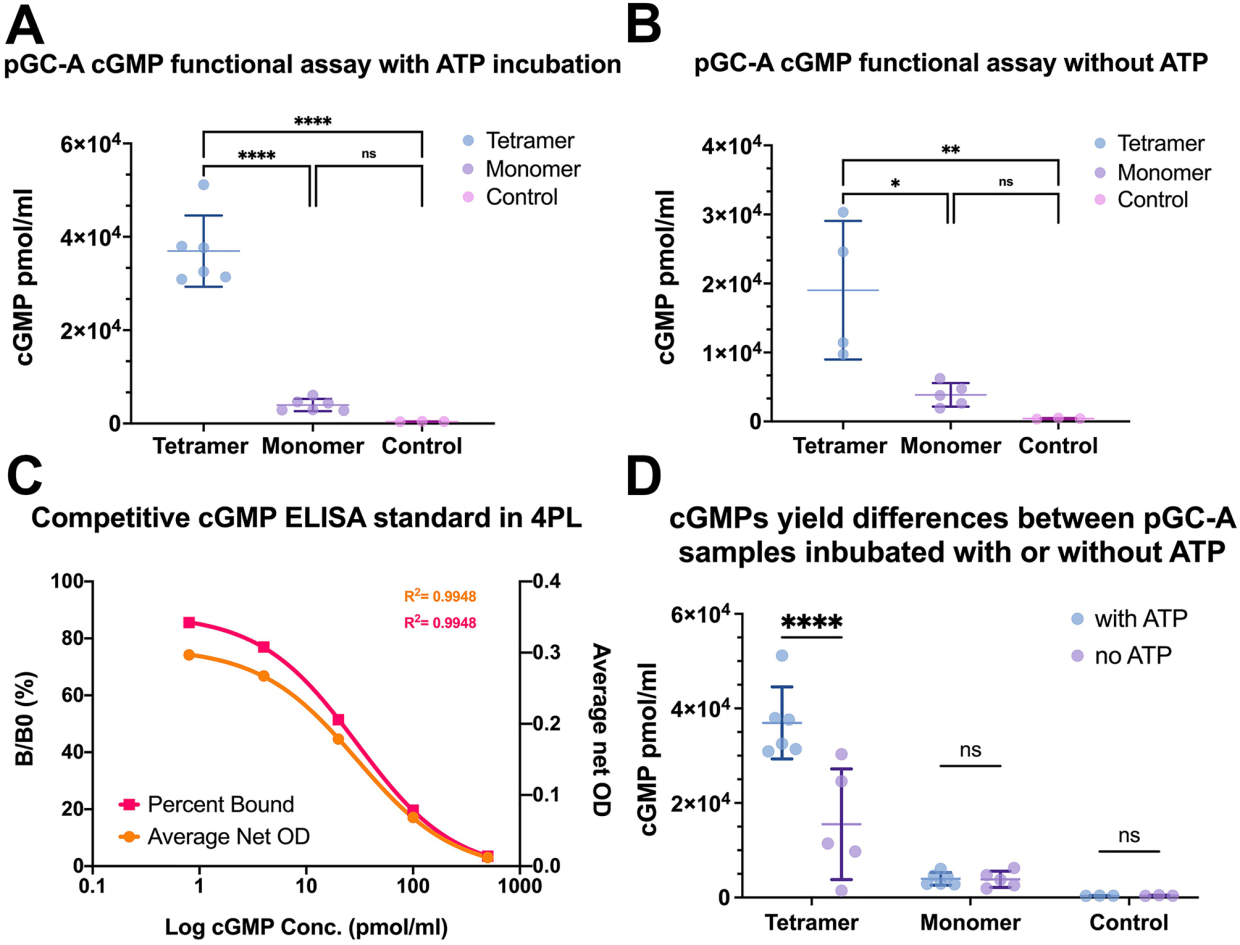
Purified full-length pGC-A in vitro functional activity test. (**A**) Functional activity for two pGC-A oligomeric states with ATP incubation. The control group was GTP and ATP in sample buffer. For activity values in units of mg of purified pGC-A, the y-axis values of pmol/mL can be converted to nmol/mg protein by multiplying by 0.00873. (**B**) Functional activity for two pGC-A oligomeric states without ATP incubation. The control group was GTP only in the sample buffer. (**C**) Competitive cGMP ELISA standard fit in four parameters logistic (4PL) curve. The left Y-axis is the B/B0 (%) value and represents the percentage of bound cGMP. The right Y-axis represents the average net optical density (OD) reading at 405 nm. Both standard curves were generated with a 95% confidence interval. (**D**) The cGMP yield differences between pGC-A oligomer samples incubated with or without ATP were analyzed. All raw data points were analyzed via the ROUT method (Q = 1%) to remove significantly impossible outlier values before data analysis. One-way ANOVA was used to determine the statistical significance between samples and control in graphs (**A**) and (**B**). Two-way ANOVA was used to determine the statistical significance in graph (**D**). Each dot represented to the sample point and plotted as mean ± standard deviation (SD). *P ≤ 0.05, **P ≤ 0.01, ****P ≤ 0.0001 and ns *P* ≥ 0.05, Not significant.

To determine if different oligomers of purified full-length pGC-A were functional and that ATP was obligated for GCD activation, two Superose 6 peak fractions, which represented tetramer and monomer sizes, were collected separately and slightly concentrated for competitive cGMP detection by ELISA. The same protein concentration of tetramer and monomer samples was established, and the samples were each split, creating two monomer and two tetramer samples that were incubated with ATP (Fig. 10A) and without ATP (Fig. 10B). The ELISA data were analyzed by GraphPad Prism9 software (GraphPad, California, USA), and the 4-parameter logistic curve fitting program^43^ was used to generate the standard cGMP concentration curve based on the cGMP percent bound (B/B0; Fig. 10C). Based on the standard cGMP curve, unknown cGMP concentrations from different samples were determined. All raw data points were first analyzed via the ROUT method (Q = 1%) to remove significantly impacting outlier values, which substantially differ from the rest of data in nonlinear regression analysis. The Q value is the maximum desired setting for the false discovery rate. A Q = 1% value represents no more than 1% of the identified outliers will be false^44^. In this experiment, one outlier value (9.4 × 10^4^ pmol/mL) from the tetramer ATP incubation group was removed before analyzing pGC-A cGMP functional assay data (Fig. 10A,B,D).

In Fig. 10A, the results revealed that the tetramer sample had a significantly higher cGMP yield when compared with the control without protein addition and the monomer sample. Additionally, there was no significant cGMP yield difference between the monomer sample and the control group. In Fig. 10B, the competitive cGMP ELISA assay analyzed the cGMP yields from purified pGC-A samples without pre-ATP incubation. The results in Fig. 10B results are very interesting as they show that the isolated pGC-A receptor can convert GTP into cGMP without prior activation by ATP. The relative activity of tetramer and monomer is similar to the results with ATP activation depicted in Fig. 10A. The major difference was that the cGMP yield in the tetramer sample was two times higher with ATP activation compared to the data without ATP activation (compare Fig. 10A with Fig. 10B).

Next, to determine whether ATP-regulated pGC-A guanylyl cyclase activity was still compatible with purified full-length pGC-A in micelle solution, as previously reported^41^, the cGMP yield differences between pGC-A oligomer samples incubated with or without ATP were further analyzed (Fig. 10D). The two-way ANOVA results showed a significant cGMP yield difference between tetramer size samples with or without ATP incubation, consistent with previous reports^41^. Additionally, there were no significant differences in monomer sample and the control group with or without ATP incubation. These significant findings corroborated the previous one-way ANOVA analysis, which indicated that the monomer sample had only minimal activity. In conclusion, the functional assay showed for the first time that purified full-length pGC-A tetramer was functional and confirmed that ATP plays a vital role in activating detergent-purified pGC-A *in* *vitro*.

## Discussion

In the present study, we established protocols for expression, purification and crystallization of human full-length pGC-A for the first time. The protein functional assay showed that purified pGC-A in solution was functional and also confirmed that ATP plays an important augmenting role for intracellular guanylyl cyclase activity in vitro.

Currently, there is available only the rat pGC-A extracellular domain structure with and without ligand ANP information^22–24^. To maximize the functional protein expression in Sf9 insect cells, we replaced the original N-terminal signal peptide with a cleavable signal peptide from influenza hemagglutinin^45^. This is the same strategy used in most G-protein-coupled receptor (GPCR) expressions in insect cells for structure determination^46,47^. Although the signal peptide was switched to the highly expressing influenza hemagglutinin, and the pGC-A DNA sequence was optimized for protein expression, the yield of functional human full-length pGC-A was quite low. The low yield may be due to protein characteristics. A previous study that expressed rat full-length pGC-A in baculovirus Sf9 insect cells reported similar modest protein production^40^. Protein sequence BLAST analysis shows that the full-length human pGC-A (UniProtKB/Swiss-Prot accession no. P16066.1) is 91.38% identical to rat pGC-A (GenBank access. no. XP_032753748.1).

Higher oligomerization of full-length pGC-A was reported in both its native expression source and recombinant expression in a mammalian cell line^27,28^. The human full-length pGC-A expressed in human embryonic kidney cell line 293 (HEK293) showed full-length pGC-A is self-associated to a possible tetrameric complex prior to ANP binding, and the truncation experiment indicates that the intracellular domain of pGC-A is necessary for self-association^28^. The partially purified full-length pGC-A from bovine adrenal cortex membranes indicates that the bovine full-length pGC-A is a disulfide linked tetramer^27^. Protein sequence BLAST shows that full-length human pGC-A is 93.12% identical to bovine pGC-A (GenBank accession no. NP_001179680.1). Our DTT incubation experiment (Fig. 9), however, clearly shows that the disulfide bond is not the critical cause of tetramer formation. In SDS-PAGE, purified pGC-A was mixed with SDS-PAGE Loading Buffer containing the reducing reagent β-mercaptoethanol, and pGC-A still showed the higher oligomer band (Fig. 3A). The purification tetramer and monomer ratio was independent of added 5 mM DTT in all purification buffers. The intracellular domain of pGC-A may interact with each other to form a higher oligomer via hydrogen bonding and hydrophobic interactions. It is possible that the tetramer in bovine is disulfide-linked, but may not be in the human full-length pGC-A.

**Figure 11:**
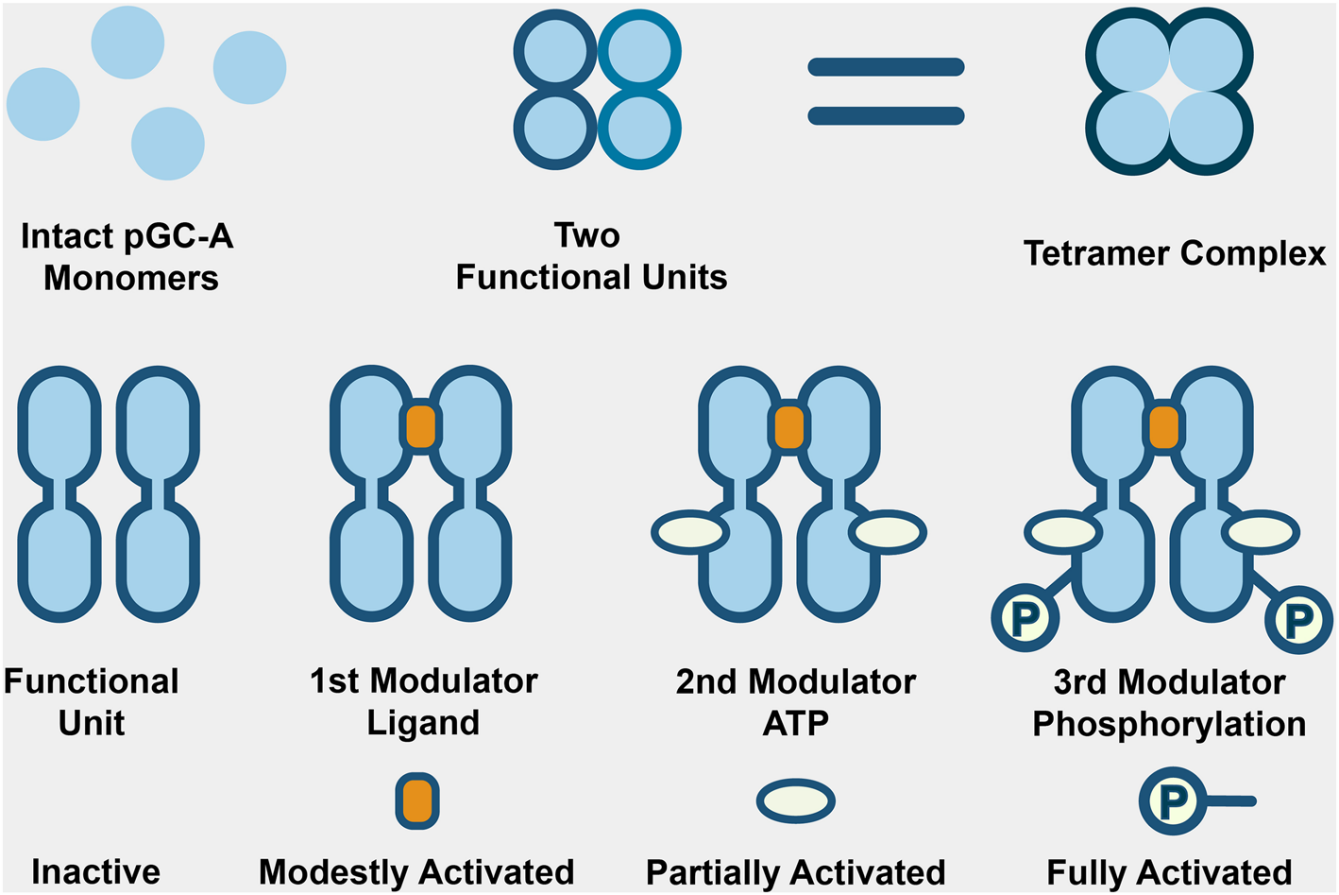
Proposed native state and three-step mechanism of full-length pGC-A. First, the full-length pGC-A forms a tetramer complex in the native state by non-covalent interactions (e.g., hydrogen bond and hydrophobic interactions). In each tetramer complex, there are two functional units, and each functional unit may represent a dimer. The narrowest part of the tetramer is the transmembrane domain. Second, the pGC-A signal transduction mechanism is not ATP-dependent. The current ATP-dependent two-step activation mechanism should instead be three-step. The first step is ligand (ANP) binding, which moderately activates the pGC-A; the second step is binding ATP, which partially boosts protein activity; the third step is the pGC-A phosphorylation, which fully activates the guanylyl cyclase.

In general, higher oligomerization is common in membrane-based receptors. For example, many GPCRs have been shown to form dimers or higher oligomers. Fluorescence labeled single molecule analysis suggested that GPCRs on the cell surface constantly self-associate and dissociate in a dynamic equilibrium manner^48^. The oligomerization of pGC-A into the tetramer state may not be caused by artificial concentration or purification. In a previous structural study of the rat pGC-A ECD, a homodimer rotation mechanism for full-length pGC-A was proposed^23^. Human full-length pGC-A may functionally work as a homodimer, but in the native form could be a tetramer complex with two functional subunits (homodimers; Fig. 11). We agree with previous discussion that the advantage of a tetramer complex could increase the local pGC-A concentration so that the tetramer complex becomes highly sensitive to very low ligand concentrations^27^. In addition, we would like to point out that all three natriuretic peptides are degraded either via clearance by natriuretic peptide receptor C or by neprilysin enzymatic degradation^49,50^. Neprilysin is a membrane protein expressed with various tissue distribution. The ANP ligand has a much higher degradation rate compared to other natriuretic peptides^49^. The higher oligomerization complex form increases the chance to repeatedly bind and release the limited ligands before ANPs get degraded.

The cGMP functional assay revealed that the purified tetramer in detergent micelle solution was functionally active (Fig. 10A,B). It also indirectly confirmed that the tetramer formation was not caused by artificial protein aggregation during protein purification but is the native functional state of the receptor. Although the monomer samples showed slightly higher cGMP yield than the control group, the cGMP yield difference between monomer and control group was not statistically significant (Fig. 10A,B). The low level of activity in the monomer fraction is likely due to the purified monomer containing a small amount of higher oligomeric states: this small amount is visible in Fig. 9 as a faint band at ~ 480 kDa in both Monomer Peak lanes and is also suggested in Fig. 7B-D by the protein absorbance (at 280 nm) that does not reach zero between the monomer and tetramer peaks in the Superose 6 size exclusion chromatography. The presence of some level of tetramer in the monomer fraction is also consistent with our results showing that the monomer forms the tetramer upon concentrating the protein (Fig. 8, Monomer Peak lane). Based on the cGMP yield results from the monomer, it can be indirectly suggested that the tetramerization of pGC-A is essential for functional activity and is a ligand-independent event. This result was consistent with previous research showing that rat pGC-A oligomerization occurred in a ligand-independent fashion^51^. The two-way ANOVA analysis showed a significant cGMP yield difference between the tetramer sample with or without ATP incubation, which is in line with earlier reports that ATP plays an important regulatory role in activating pGC-A^41,52^.

The current pGC-A signal transduction mechanism proposes an ATP-dependent two-step ordered process. First, ATP binds to the ATP-binding pocket to activate the ATP-dependent allosteric step, which partially activates the GCD and triggers a conformational change; second, the phosphorylation of the potential ATP-binding domain fully activates guanylyl cyclase^41,42^. However, in our cGMP functional assay, purified tetramer size protein still showed significant cGMP yield without ATP incubation (Fig. 10B), but the cGMP yield was increased dramatically with ATP incubation (Fig. 10A). Our findings suggested that ATP may not be critical for initiating pGC-A activation, as suggested by previous reports^40^. Binding the ligand will moderately activate guanylyl cyclase function. One report revealed that activation of the guanylyl cyclase was ATP-independent^53^. Our functional assay results with purified human pGC-A were consistent with previous research with rat pGC-A from the cell membrane fraction, which also showed that ATP accelerated the guanylyl cyclase activity^52,54^. However, our functional assay results (Fig. 10B) also indicated that the ligand itself will activate pGC-A even without ATP^53^. Collectively, our study suggested that three modulators modulate different activation levels in full-length pGC-A (Fig. 11). The first level is ligand binding to the ECD, which moderately activates the pGC-A; the second level is binding ATP to the ATP-binding pocket at the ICD, which acts as an augment modulator and boosts protein activity; the third level is pGC-A phosphorylation, which fully activates the guanylyl cyclase functionality.

We expect that structures of full-length pGC-A could confirm models that have been previously proposed. For example, mutational and kinetic analyses by Edmund et al. 2019^54^ led to their proposal that the dynamic hydrophobic core model of canonical protein kinases also explains how ATP binding to the protein kinase-like domain (PKLD) of pGC-A affects its guanylyl cyclase activity. This model could be confirmed upon comparison of structures of full-length pGC-A (wild-type and/or mutant) with and without non-hydrolyzable ATP analogs. Also, Ottemann et al. 1999^55^ summarized five possible mechanistic models for transmembrane signal transduction in oligomeric receptors that contain a single transmembrane domain per subunit. To distinguish between the five models in the dimeric aspartate receptor from *Salmonella typhimurium*, this study applied strategically placed nitroxide spin labels and electron paramagnetic resonance, which revealed a ~ 1 angstrom movement of one transmembrane helix relative to the other transmembrane helix, consistent with a Piston model of transmembrane signaling. Structural evidence of this movement was later supported by a piston-like displacement of the periplasmic helix α4b′ in the aspartate receptor, as seen by Mise 2016^56^, upon comparison of crystal structures of the periplasmic domain in the presence and absence of aspartate (PDB 4Z9H/4Z9I and 4Z9J, respectively). Other transmembrane proteins that exist as tetramers include several ion channels, which possess more than one transmembrane domain per subunit. For the tetrameric ionotropic glutamate receptor (rat GluA2), agonist binding (PDB 4U4F) causes a tightening of the extracellular dimer-dimer interface, which leads to a spreading apart of each subunit’s transmembrane domain relative to the other subunits^57^, analogous to the See-Saw mechanism described by Ottemann et al. 1999^55^. A similar See-Saw mechanism could be accomplished in pGC-A but with fewer transmembrane domains compared to the ion channels. The actual mechanism of transmembrane signaling in pGC-A is still unclear and may be revealed in future structures of the full-length pGC-A with and without the natriuretic peptide ligand.

In future work, we would like to solve the atomic-resolution structure of full-length pGC-A. The number of proteins packing in the crystal unit cell could indirectly prove if full-length pGC-A is a tetramer form or other types. Tetramer structures might also reveal why a dimer was not predominant in purified pGC-A (Figs. 7B-D); for example, the dimer-dimer interfaces that are revealed in the tetramers may have properties that, as a dimer, would appear unstable in the surrounding environment. Also, revealing the intracellular domain of full-length pGC-A is critical to understanding the protein signaling transduction from ligand binding in the extracellular domain to activation of the guanylyl cyclase. Future studies must be conducted to determine how exactly ATP interacts with the intracellular protein kinase-like domain (PKLD). There are still many interesting structural-based studies that could be done. So far, there is yet no available human guanylyl cyclase structure. The structural information from full-length pGC-A could fill this gap. In addition, one functional study indicates that the ANP-stimulated guanylyl cyclase reaction is dependent on ATP as a cofactor^39^. PKLD mediates both the regulatory action of ATP for signal transduction and modulation of NPR-A hormone affinity^39^. In general, the ANP/pGC-A/cGMP pathway responds to control of renal, endocrine and cardiovascular homeostasis. Thus, pGC-A is an important membrane receptor in medical research for drug development. Knowing the structure of full-length pGC-A is only a starting point for many new related studies.

## Methods

### Construction of the pFastBac1-pGC-A expression vector

The Invitrogen Bac-to-Bac BEVS protocol was followed to express full-length human pGC-A. The plasmid construct pFastBac1-pGC-A (Addgene #186626) was designed to allow expression in Sf9 insect cells of pGC-A (amino acids 33–1061 of NCBI Reference Sequence NP_000897.3). The gene encoding the pGC-A protein was optimized by GenScript for expression in insect cells using GenScript's OptimumGene algorithm and cloned by GenScript into the *Bam*HI and *Hin*dIII sites of the insect cell donor vector pFastBac1. The final vector was sequence-verified by GenScript. The protein diagram is shown in Supplementary Fig. S1A, the DNA diagram is shown in Supplementary Fig. S1B, and the complete protein and DNA sequences are in Supplementary Figs. S2A and B, respectively. The native signal peptide sequence (amino acids 1–32) was replaced with a cleavable N-terminal influenza virus hemagglutinin signal peptide^45^. A tobacco etch virus (TEV) protease cleavable His10-tag was added between the signal peptide and the pGC-A sequence.

### Small-scale cultures for optimization of the virus MOI and harvest days

Insect cell culture, recombinant bacmid generation, and recombinant baculovirus generation and amplification are detailed in the Supplementary Methods. During the screening test, a multiplicity of infection (MOI) of 0, 3, 5, 10 was used to transfect 4 mL of Sf9 insect cell culture. MOI is the ratio of the number of virus particles to the number of cells in cell culture. A 4 mL volume of culture was collected after post-transfection at 48, 72, and 96 h. A 100 μL volume of cell culture was pelleted and lysed with 50 μL of SDS-PAGE Loading Buffer (1X XT Sample Buffer [Bio-Rad #161-0791], 715 mM β-mercaptoethanol). All samples were heated up at 96 °C for 10 min before loading on SDS-PAGE. Samples were analyzed by SDS-PAGE on 4–12% acrylamide Bis–Tris gels (Invitrogen # NP0329BOX) in 1X MOPS-SDS running buffer (Invitrogen, # NP0001). For western blotting, the primary antibody, anti-pGC-A, was purchased from R&D Systems (# MAB4860); the secondary antibody, HRP-conjugated anti-mouse IgG, was procured from Jackson ImmunoResearch (# 515–035-062). Original blots/gels are in Supplementary Fig. S7.

### Whole-cell activity assay for full-length pGC-A expressed in Sf9 cells

The cGMP Complete ELISA Kit (Enzo Life Sciences, # ADI-901–164) was employed to measure the whole-cell activity assay. The exact cell preparation and ligand incubation process have been described previously^30,58^. Both transfected and wildtype Sf9 insect cells were grown for 3 days in the incubator. Later, insect cells were pelleted via centrifugation and suspended in fresh Sf-900 III SFM medium (Gibco, # 12,658,027). Various concentrations (1 × 10^−5^ M, 1 × 10^−6^ M, 1 × 10^−7^ M, 1 × 10^−8^ M) of ligand MANP (Bachem, customized peptide) with 5 × 10^−3^ M of nonspecific phosphodiesterase inhibitor 3-isobutyl-1-methylxanthine (IBMX; Sigma, # I5879-250MG) were added and incubated with insect cells for 20 min. Then, cells were pelleted and lysed with the lysis buffer (sodium acetate buffer containing proteins and sodium azide) provided in the assay kit. The exact ELISA procedure is described in the kit. Briefly, the lysed samples, standards, and controls were added to a 96-well microtiter plate coated with goat anti-rabbit IgG antibody (Enzo Life Sciences, # ADI-80–0060). The solution of cGMP conjugated to alkaline phosphatase was then added, followed by a solution of rabbit polyclonal antibody to cGMP. After incubation for 2 h at room temperature with shaking at 500 rpm, the plate was washed three times with wash buffer. A substrate solution of p-nitrophenyl phosphate was added to the plate. Then, the plate was incubated at room temperature for 1 h without shaking. The stop solution was added, and the plate was read at 405 nm, with a blank against the substrate.

### Purified full-length pGC-A in vitro functional activity test

The cGMP Complete ELISA Kit instructions were followed to measure the purified pGC-A activity with a few modifications. Purified pGC-A protein peak fractions (tetramer and monomer size) were collected separately. The same amount of concentrated peak fractions was mixed with human ANP (hANP; Sigma, # A1663-0.5MG) and GTP (Sigma, # G8877-25MG) and incubated overnight on ice. Depending on the experimental setting, ATP (Sigma, # A1852-1VL) was also added during the incubation. To maintain hANP stability and integrity, 5 mM DTT was removed in the final Superose 6 size exclusion column, and no reducing reagent was added to the protein sample buffer.

In this specific assay experiment, both tetramer and monomer size fraction samples were concentrated to 0.36 mg/mL. To prepare the non-ATP incubated samples, 35 μL of each protein sample was mixed with 5 μL of hANP (5 mg/mL) and 35 μL of GTP (13.7 mg/mL). The total volume of the non-ATP incubated sample was 75 μL. For the non-ATP incubated control sample, 35 μL of GTP (13.7 mg/mL) was mixed with 40 μL of Protein Purification Buffer (50 mM HEPES–NaOH, pH 7.5, 150 mM NaCl, 10% glycerol, 0.05% DDM, 0.01% CHS). For preparing the ATP incubated samples, 35 μL of each protein sample was mixed with 5 μL of hANP (5 mg/mL), 35 μL of GTP (13.7 mg/mL) and 35 μL of ATP (26.4 mg/mL). The total volume of the ATP incubated sample was 110 μL. For the ATP incubated control sample, 35 μL of GTP (13.7 mg/mL) and 35 μL of ATP (26.4 mg/mL) were mixed with 40 μL of Protein Purification Buffer. All prepared samples were incubated on ice in the cold room (4 °C) and subjected to overnight incubation.

After overnight incubation, all ELISA standards, controls, and samples were prepared and diluted with Protein Purification Buffer. Then, 100 μL of the standards, controls, and samples were added to a 96-well microtiter plate coated with goat anti-rabbit IgG antibody (Enzo Life Sciences, # ADI-80–0060). Next, a cGMP solution conjugated to alkaline phosphatase was then added, followed by the anti-cGMP reagent of rabbit polyclonal antibody. After 2 h of incubation at room temperature with shaking at 500 rpm, the ELISA plate was washed three times using the wash buffer (Tris buffered saline containing detergents). The p-nitrophenyl phosphate substrate solution was added to the ELISA plate. The plate was incubated at room temperature for 1 h without shaking. Then, a stop solution (trisodium phosphate solution) was added, and the ELISA plate was read at 405 nm, with a blank against the substrate.

### Large-scale pGC-A expression and purification

Insect cells were grown and scaled up in commercial Sf-900 III SFM serum-free medium in a 27 °C incubator. A culture with 1.5 L of 2 × 10^6^ Sf9 insect cells/mL was transfected with generated pGC-A virus with a MOI of 10. Seventy-two hours post-infection, the cells were harvested by centrifugation at 2,000 × *g* for 10 min at 4 °C. Harvested cells were suspended in ice-chilled lysis buffer (50 mM HEPES–NaOH, pH 7.5, 10 mM MgCl_2_, 20 mM KCl, 0.01 mg/mL deoxyribonuclease I [Sigma, # 11284932001], 10% glycerol) and lysed on an ice-water bath via sonication (1 s pulse on and 2 s pulse off, for a total of 3.5 min at 40% power; sonicator model Branson Sonifier SFX550). The cellular membrane fraction was collected using a 45-Ti rotor at 45,000 rpm using a Beckman Coulter ultracentrifuge for 30 min at 4 °C. Then, a 15 mL glass KIMBLE tissue homogenizer was used to resuspend the cellular membrane fraction with hypertonic buffer (50 mM HEPES–NaOH, pH 7.5, 10 mM MgCl_2_, 20 mM KCl, 5 mM DTT, 1 M NaCl, 10% glycerol) on ice; subsequently, the solubilization buffer (50 mM HEPES–NaOH, pH 7.5, 150 mM NaCl, 5 mM DTT, 10% glycerol) was used to wash the membrane fraction with a tissue homogenizer on ice. The washed membrane fraction was pelleted down in a Beckman Coulter 70-Ti at 45,000 rpm for 30 min at 4 °C. The washed and pelleted membrane fraction was solubilized with 0.75% DDM (Glycon Biochemicals, # D97002-C) and 0.15% CHS (Sigma, # C6512-5G) for 1 h on ice. The solubilized membrane solution was centrifuged in 70-Ti at 45,000 rpm for 30 min at 4 °C. The solubilized membrane supernatant was incubated with 300–400 µL of pre-equilibrated Ni^2+^-charged immobilized metal affinity chromatography resin (Bio-Rad, # 7800801) for 30 min at 4 °C. The resin was washed with 30 column volumes (CVs) of buffer A (50 mM HEPES–NaOH, pH 7.5, 500 mM NaCl, 5 mM DTT, 50 mM imidazole–HCl, 10% glycerol, 0.05% DDM, 0.01% CHS), 15 CVs of buffer B (buffer A with 90 mM imidazole–HCl) and eluted with 10 CVs of buffer C (buffer A with 500 mM imidazole–HCl). The eluted sample was collected and concentrated in a 15 mL 100 kDa cutoff Amicon Ultracel-100 regenerated cellulose membrane concentrator (EMD Millipore, # UFC910024) at 3,000 × *g* at 4 °C. The concentrated elution fraction (300–500 µL volume) was injected into a pre-equilibrated (50 mM HEPES–NaOH, pH 7.5, 150 mM NaCl, 5 mM DTT, 10% glycerol, 0.05% DDM, and 0.01% CHS) Superose 6 Increase 10/300 GL column (Cytiva, # 29091596). The ÄKTA pure chromatography system used with the Superose 6 column at a flow rate of 0.45 mL/min for a total of 1 CV (24 mL) run at 4 °C. High resolution clear-native PAGE is detailed in the Supplementary Methods.

### Initial protein crystallization screening

Purified pGC-A protein from the Superose 6 chromatography runs was concentrated to 2 mg/mL using a 100 kDa cutoff ultrafiltration concentrator by centrifugation at 12,000 × *g* at 4 °C. To reduce the possibility of the protein directly reaching the precipitation zone (where an amorphous precipitate is formed), we screened using 80% concentration of the original MemGold2 HT-96 kit (Molecular Dimensions, # MD1-64) concentration of precipitants. The crystallization conditions were manually screened in 24-well plates by the hanging drop (vapor diffusion) method. To avoid protein degradation, the plates were set up and incubated in a cold room at 4 °C. First, 1 µL of concentrated protein sample was mixed with 1 µL of 20% diluted MemGold2 HT-96 screening precipitant solution on a glass cover slide. Then, glycerol was added to the precipitant solution in each well (a final total of 10% glycerol concentration) to compensate for the glycerol brought into the crystallization drop by the protein solution; the glass cover slide was flipped and sealed back onto the well. The plates were checked 3 and 5 days after setup. During the initial crystallization screening, promising crystallization conditions were identified based on the formation of crystalline structures that showed a signal in the SONICC (Second Order Nonlinear Imaging of Chiral Crystals) imaging system^59^. Images of drops in the 24-well plates were captured under visible light, SONICC Hi-Res, and UV TPEF Hi-Res modes.

### Crystallization optimization screening

The best initial crystallization condition (0.32 M ammonium sulfate, 0.08 M MES buffer, pH 6.5, 8% PEG 3350) was optimized as follows. Both the salt (ammonium sulfate) and the precipitant (PEG 3350) were used to create narrow step gradient screenings in a 24-well plate format. The protein concentration was 1.25 mg/mL. In the AB rows, the concentration of PEG 3350 was varied from 5 to 10% with 0.08 M MES buffer, pH 6.5, and 0.32 M ammonium sulfate. In the CD rows, the concentration of ammonium sulfate was varied from 0 M to 0.4 M in 0.08 M MES, pH 6.5, and 8% PEG 3350. The method for confirming that pGC-A formed protein crystals is described in the Supplementary Methods.

### Protein crystal diffraction and data collection

A 1.25 mg/mL concentration of pGC-A was crystallized using the following precipitant (0.32 M ammonium sulfate, 0.08 M MES, pH 6.5, 7–12% PEG 3350). Needle-shaped crystals from 96 crystallization drops (four plates) were harvested and placed into 0.65 mL Eppendorf tubes and shipped to APS (Argonne National Laboratory, Chicago, IL) within a temperature-controlled 4 °C container. Embedding of crystals in lipidic cubic phase (LCP) was performed as described previously^33^. Briefly, 20 μL of lipid-sample mixture were prepared by mixing 8 μL of the crystallization precipitant solution with 12 μL molten monoolein 9.9 MAG (1-oleoyl-*rac*-glycerol Sigma, # M7765-1G) in a glass dual-syringe lipid mixer until a homogeneous transparent LCP was formed. Then, the crystal slurry was gently centrifuged at 100 × *g* for 20 min at 4 °C to pellet the crystals. After removing the supernatant, the pelleted crystals (5 μL) were loaded into a 100 μL glass syringe and mixed with previously prepared lipid/precipitant mixture in a glass dual-syringe lipid mixer. To prevent the sample from heating and to ensure a homogeneous distribution of the crystals within the LCP phase, mixing was carried out in a total of 15 min by alternating series of 1 min mixing (ON) followed by 1 min rest (OFF). Then, the LCP embedded crystal sample was loaded into the LCP injector developed by Uwe Weierstall^32^, with a 50 μm glass capillary nozzle. The sample flow rate was kept constant to 15 nL/min during data collection, and the LCP-embedded crystal sample was delivered to the synchrotron X-ray beam at room temperature.

Serial data collection was performed on the GM/CA 23-ID-D beamline at APS using the same experimental setup previously described^33^. Data collection was performed in a shutterless mode at a repetition rate of 100 Hz (10 ms exposure time/image), which was achieved using the continuous readout of the PILATUS3 6 M detector. X-ray diffraction data were collected at a photon energy of 11.5 keV (1.07 Å) and a beam size of 16 × 10 μm (H × V). The sample-to-detector distance was fixed at 300 mm. A total of 270,000 frames were recorded on the detector, five of which were identified as hits. Diffraction patterns were indexed by the CrystFEL program^34^.

## Supplementary Information

Supplementary Information.

## Data Availability

All data generated or analyzed during this study are included in this published article (and its Supplementary Information files). The plasmid pFastBac1-pGC-A is available in GenBank under accession number OL860945.
