# Supplementary material for: Purification, characterization, and preliminary serial crystallography diffraction advances structure determination of full-length human particulate guanylyl cyclase A receptor
Source: Sci Rep. 2022 Jul 12;12:11824. doi: 10.1038/s41598-022-15798-z (PMC9276669; doi:10.1038/s41598-022-15798-z)
Supplement: Supplementary file 1 — Supplementary Information. [file 41598_2022_15798_MOESM1_ESM.pdf]

Supplementary Information for

Purification, characterization, and preliminary serial crystallography diffraction advances structure determination of full-length human particulate guanylyl cyclase A receptor

Shangji Zhang, Debra T. Hansen, Jose M. Martin-Garcia, James D. Zook, Shuchong Pan,
Felicia M. Craciunescu, John C. Burnett, Jr., Petra Fromme

Supplementary Methods

Insect cell culture. *Spodoptera frugiperda* (Sf9) cells were purchased from Thermo Fisher (# 12659017). Cells were seeded at a density of 0.6 × 10^6 viable cells/mL. Insect cells were grown in suspension flasks with Sf-900 III SFM medium (Gibco, # 12658027). Seeded cells were incubated at 27°C with shaking at 130 rpm on an orbital shaker and passaged when the cell density reached 2 × 10^6 viable cells/mL. Cell counting was performed using the Thermo Countess II cell counter. During each passage, 10 µL of the suspension cell culture was mixed with 10 µL Trypan Blue stain (from the Countess Cell Counting Chamber Slides, Thermo Fisher # C10228) to measure the cell density and viability. The cells were sub-cultured at the same seeding density until reaching the 30th passage. Then, another vial of frozen cell stock was used for a new passage.

Recombinant bacmid generation. To generate the pGC-A recombinant bacmids, the Invitrogen Bac-to-Bac BEVS protocol was followed. Purified 50 µL pFastBac1-pGC-A plasmids were transformed into *Escherichia coli* DH10Bac competent cells (Invitrogen, # 10361-012). For each transformation, one vial of commercial DH10Bac competent cells was thawed on ice and mixed with 1 ng of purified pFastBac1-pGC-A plasmid with a gentle mix. The mixed cells were cooled on ice for 30 min. The chilled cell-plasmid mixture was heat-shocked for 45 s at 42°C and immediately placed back on ice for 2 min. Then, 900 µL of room temperature SOC medium (from the competent cells kit) was added to the cell-plasmid mixture, and the mixture was incubated at 37°C for 4 h with shaking at 225 rpm. Then, 100 µL transformed *E. coli* DH10Bac cells were grown on the X-gal LB plate (containing 50 µg/mL kanamycin, 7 µg/mL gentamicin, 10 µg/mL tetracycline, 100 µg/mL Bluo-gal, and 40 µg/mL isopropyl β-D-1-thiogalactopyranoside) for 48 h at 37°C. The single, well-isolated white colonies were picked for whole-cell colony Polymerase Chain Reaction (PCR).

For colony PCR, well-isolated white colonies were picked with a 20 µL pipet tip and transferred to a PCR tube with 20 µL molecular biology grade water (Corning, # 46000CV). Well-mixed samples were heated for 10 min at 90°C and centrifuged at 13,000 x *g* for 5 min to collect the supernatant. Then, 10 µL of colony supernatant solution was mixed with 0.3 µL of 10 mM Deoxynucleotide Solution Mix (New England Biolabs, # N0447S), 0.6 µL 100 µM forward primer BACM13F (5'-CCCAGTCACGACGTTGTAAAACG-3'), 0.6 µL 100 µM reverse primer BACM13R (5'-AGCGGATAACAATTTCACACAGG-3'), 6 µL iProof High-Fidelity polymerase (Bio-Rad, # 1725301), and 23.28 µL molecular grade water. The initial denaturation time was 1 min at 98°C and followed with the repeated cycles: 10 s denaturation time at 98°C; 45 s annealing time at 50°C; 6 min extension time at 72°C. The total PCR had 35 cycles.

Recombinant baculovirus generation and amplification. The PCR-confirmed whole-cell colonies were inoculated in 4 mL LB medium (containing 50 µg/mL of kanamycin,7 µg/mL of gentamicin, and 10 µg/mL of tetracycline) and cultured overnight at 37°C with shaking at 225 rpm in an orbital shaker. The recombinant bacmid DNA was purified with the Invitrogen PureLink HiPure Plasmid Miniprep Kit (Invitrogen, # K210002). The following solutions and column were from the kit. In summary, overnight cell culture was pelleted and resuspended with 400 µL resuspension buffer (50 mM Tris-HCl, pH 8.0, 10 mM EDTA) with 20 mg/mL RNase A in a 1.5 mL microcentrifuge tube. Then, 400 µL of lysis buffer (0.2 M NaOH, 1% [wt/vol] SDS)

was added, and the tube was gently inverted 5 times. Next, 400 µL precipitation buffer (3.1 M potassium acetate, pH 5.5) was added, and the tube was repeatedly inverted until the mixture was homogenous. The tube was centrifuged for 10 min at 17,000 x *g*. Finally, 1 mL of the supernatant was transferred to an equilibrated column (equilibrated with 2 mL of equilibration buffer [0.1 M sodium acetate, pH 5.0, 0.6 M NaCl, 0.15% (vol/vol) Triton X-100]). The column was washed twice with 2.5 mL wash buffer (0.1 M sodium acetate, pH 5.0, 825 mM NaCl), and recombinant bacmid was eluted with 0.9 mL elution buffer (100 mM Tris-HCl, pH 8.5, 1.25 M NaCl). Then, 0.63 mL isopropanol was added to the eluted solution. The inverted mixed tube was chilled on ice for 10 min. The sample was centrifuged at 17,000 x *g* for 20 min at 4°C. After discarding the supernatant, 1 mL of 70% ethanol was added to the tube. The inverted tube was further centrifuged at 17,000 x *g* for 5 min at 4°C. After discarding the supernatant, the bacmid pellet in the tube was air-dried for 10 min. Finally, 40 µL of TE buffer (10 mM Tris-HCl, pH 8.0, 0.1 mM EDTA) was added to gently resuspend the purified bacmid.

The purified 40 µL bacmid DNA (50-100 ng/µL) was mixed with 100 µL fresh Sf-900 III SFM medium via finger flick of the tube. Next, 8 µL Cellfectin II Reagent (Thermo Scientific, # 10362100) was mixed with insect cell fresh media via vortexing. Then, 108 µL transfecting reagent was added to the bacmid solution with finger tapping, followed by incubation for 30 min. The ratio of the transfection mixture is 1 µg DNA/8 µL Cellfectin II reagent. The transfected mixture was added to 1 mL (1×10^6 cell/mL) insect cell culture in sterile 24-well deep well plates (10.4 mL per well, V-bottom, square well; Thomas Scientific, # 504361) and incubated for 7 days at 27°C with shaking at 130 rpm to generate the Passage 1 (P1) virus stock. After 7 days of virus generation, P1 culture was centrifuged for 5 min at 1,000 x *g*. The supernatant, which contained the P1 recombinant virus, was stored and used to generate the Passage 2 (P2) virus stock. Then, 200 µL of P1 virus supernatant was added to 4 mL (0.5×10^6 cell/mL) insect cell culture in a 24-well plate and incubated for 3 days at 27°C with shaking at 130 rpm to generate the P2 virus stock. After 3 days of virus generation, the P2 culture was centrifuged for 5 min at 1,000 x *g*. The supernatant, which contained the P2 virus, was stored and used to generate the Passage 3 (P3) virus stock. Subsequently, 500 µL of P2 virus supernatant was added to 30 mL (3×10^6 cell/mL) insect cell culture and incubated for 4 days at 27°C with shaking at 130 rpm to generate the final P3 virus stock. All cell pellets from three virus passages were saved for western blot protein expression analysis. The P3 viral concentration was measured before storing in liquid nitrogen. The virus titer was determined based on viable transfected cell diameter size change and statistically analyzed and calculated by the method of moment[60]. The precise virus titer protocol was previously published[61], and the exact steps were followed during the titer determination experiment. Briefly, 2 mL of different serial dilutions of virus stock and one mock medium were prepared. Then, each virus dilution (2 mL) was added to 20 mL of 2×10^6 cells/mL Sf9 cells with 95% viability and incubated at 27°C with shaking at 130 rpm on an orbital shaker for 24 h. Subsequently, 1 mL of each transected cell culture was used to measure the cell diameter via the Beckman Vi-CELL counter in duplicate mode. Then, the measured cell diameter data were analyzed, statically calculating the virus titer using the method of moment[61].

Confirmation that pGC-A formed protein crystals. To confirm if the needle-shaped crystals were composed of the pGC-A protein, 3-week-old crystallization drops were collected via pipetting the drop content of five crystallization drops into a PCR tube. Collected crystals were pelleted down by centrifugation and washed with crystallization precipitant solution. The protein crystals were pelleted twice at a maximum 17,000 x *g* for 10 minutes in 4°C. Samples of the

centrifugation supernatant, the washed crystal pellets, as well as a sample from the combined crystallization drop were each mixed with SDS-PAGE Loading Buffer in 1:1 volume ratio. To partially preserve the multimers in the protein crystals, the prepared SDS samples were directly loaded into the SDS-PAGE without heating. Western blot analysis was used to detect the pGC-A signal. For the western blot, the primary antibody, anti-pGC-A, was ordered from R&D Systems (# MAB4860) and the secondary antibody, HRP-conjugated anti-mouse IgG, was ordered from Jackson ImmunoResearch (# 515-035-062).

High Resolution Clear-Native PAGE. High resolution clear native polyacrylamide gel electrophoresis (hrCN-PAGE) was performed as described by Wittig *et al.*[62] Briefly, 12 μL of concentrated protein was mixed with 12 μL of 2X Native PAGE Sample Loading Buffer (100 mM sodium chloride, 100 mM imidazole-HCl, 4 mM 6-aminohexanoic acid, 10% glycerol, 2 mM EDTA, pH 7.0) immediately before loading the sample onto the native polyacrylamide gel. During the running buffer preparation, 0.05% (DDM) was added to the cathode buffer (50 mM Tricine-NaOH, 7.5 mM imidazole-HCl, pH 7.0). The cathode buffer and the anode buffer (25 mM imidazole-HCl, pH 7.0) were freshly prepared for single usage. The 4-16% native polyacrylamide gel was from Thermo Scientific (Invitrogen, # BN1002BOX). Native gel electrophoresis was performed in an ice-chilled basket, and hrCN-PAGE was run at a constant 150 V for a total of 2 h.

Supplementary Figures

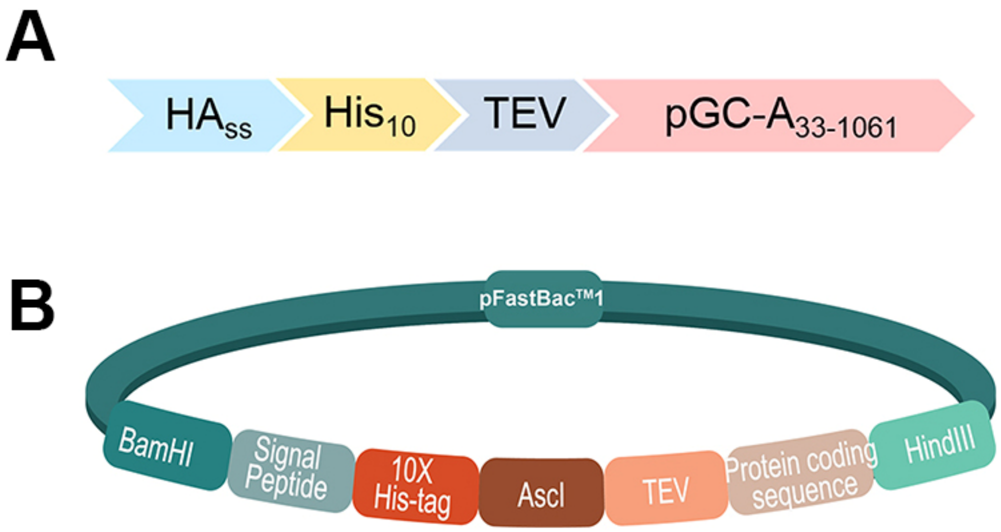

Supplementary Figure S1. Schematic of the pGC-A donor vector and protein diagram. (**A**) Protein diagram. (**B**) The full-length pGC-A donor vector is 7,877 bp. Abbreviations: HA_{ss}, hemagglutinin signal sequence; TEV, tobacco etch virus protease cleavage site.

A

```
LOCUS       Translation\of\pFastBac1-GCA        1065 aa                          2-MAY-2018
SOURCE
  ORGANISM
FEATURES             Location/Qualifiers
     Site            17..26
                     /vntifkey="264"
                     /label=His10
     Site            30..36
                     /vntifkey="221"
                     /label=TEV\protease\cleavage\ENLYFQ*G
     Region          37..1065
                     /vntifkey="1000"
                     /label=pGC-A\NP_000897.3\aa33-1061
     Region          1..16
                     /vntifkey="200"
                     /label=HA\signal\peptide
     misc_feat       37..477
                     /vntifkey="273"
                     /label=soluble\fragment\NP_000897.3\aa33-473
ORIGIN
        1 mktiialsyi fclvfahhhh hhhhhhgape nlyfqggnlt vavvlplant sypwswarvg
       61 pavelalaqv karpdllpgw tvrtvlgsse nalgvcsdta aplaavdlkw ehnpavflgp
      121 gcvyaaapvg rftahwrvpl ltagapalgf gvkdeyaltt ragpsyaklg dfvaalhrrl
      181 gwerqalmly ayrpgdeehc fflveglfmr vrdrlnitvd hlefaeddls hytrllrtmp
      241 rkgrviyics spdafrtlml laleaglcge dyvffhldif gqslqggqgp aprrpwergd
      301 gqdvsarqaf qaakiitykd pdnpeylefl kqlkhlayeq fnftmedglv ntipasfhdg
      361 lllyiqavte tlahggtvtd genitqrmwn rsfqgvtgyl kidssgdret dfslwdmdpe
      421 ngafrvvlny ngtsqelvav sgrklnwplg ypppdipkcg fdnedpacnq dhlstlevla
      481 lvgslsllgi livsffiyrk mqlekelase lwrvrwedve psslerhlrs agsrltlsgr
      541 gsnygslltt egqfqvfakt ayykgnlvav krvnrkriel trkvlfelkh mrdvqnehlt
      601 rfvgactdpp nicilteycp rgslqdilen esitldwmfr ysltndivkg mlflhngaic
      661 shgnlkssnc vvdgrfvlki tdyglesfrd ldpeqghtvy akklwtapel lrmasppvrg
      721 sqagdvysfg iilqeialrs gvfhvegldl spkeiiervt rgeqppfrps lalqshleel
      781 gllmqrcwae dpqerppfqq irltlrkfnr enssnildnl lsrmeqyann leelveertq
      841 ayleekrkae allyqilphs vaeqlkrget vqaeafdsvt iyfsdivgft alsaestpmq
      901 vvtllndlyt cfdavidnfd vykvetigda ymvvsglpvr ngrlhaceva rmalalldav
      961 rsfrirhrpq eqlrlrigih tgpvcagvvg lkmpryclfg dtvntasrme sngealkihl
     1021 ssetkavlee fggfelelrg dvemkgkgkv rtywllgerg sstrg
```

Supplementary Figure S2. Expression clone detail. (**A**) Protein sequence. (**B**) Plasmid DNA sequence. The sequences are available in GenBank accession number OL860945.

B

```
LOCUS       pFastBac1-GCA        7877 bp    DNA     circular SYN  2-MAY-2018
SOURCE
  ORGANISM
FEATURES             Location/Qualifiers
     rep_origin      2..457
                     /direction=RIGHT
                     /vntifkey="33"
                     /label=f1\ori
                     /note="f1 bacteriophage origin of replication; arrow indicates direction of (+) strand synthesis"
     promoter        484..588
                     /gene="bla"
                     /vntifkey="30"
                     /label=AmpR\promoter
     CDS             589..1449
                     /codon_start=1
                     /gene="bla"
                     /product="beta-lactamase"
                     /vntifkey="4"
                     /label=ampicillin-R
                     /note="confers resistance to ampicillin, carbenicillin, and related antibiotics"
     rep_origin      1620..2208
                     /direction=RIGHT
                     /vntifkey="33"
                     /label=pUC\ori
                     /note="high-copy-number ColE1/pMB1/pBR322/pUC origin of replication"
     mobile_element  2513..2737
                     /mobile_element_type="transposon:Tn7"
                     /vntifkey="21"
                     /label=Tn7R
                     /note="Unknown feature type:mobile_element  mini-Tn7 element (right end of the Tn7 transposon)"
     CDS             complement(2804..3337)
                     /codon_start=1
                     /gene="aacC1"
                     /product="gentamycin acetyltransferase"
                     /vntifkey="4"
                     /label=gentamycin-R\(GmR)
                     /note="confers resistance to gentamycin"
     promoter        complement(3526..3554)
                     /gene="intI1 (promoter lies within the coding sequence)"
                     /vntifkey="30"
                     /label=Pc\promoter
                     /note="class 1 integron promoter"
     promoter        3906..3997
                     /gene="polh from Autographa californica"
                     /vntifkey="30"
                     /label=polyhedrin\promoter
                     /note="promoter for the baculovirus polyhedrin gene"
     polyA_signal    7367..7501
                     /vntifkey="25"
                     /label=SV40\poly(A)\signal
                     /note="SV40 polyadenylation signal"
     mobile_element  7530..7695
                     /mobile_element_type="transposon:Tn7"
                     /vntifkey="21"
                     /label=Tn7L
                     /note="Unknown feature type:mobile_element  mini-Tn7 element (left end of the Tn7 transposon)"
     primer          3922..3939
                     /vntifkey="27"
                     /label=PRIMER\POLYHEDF
     primer          complement(7267..7286)
                     /vntifkey="27"
                     /label=PRIMER\PFASTBACR
     primer          complement(7404..7423)
                     /vntifkey="27"
                     /label=PRIMER\SV40PAR
     5'clip          4034..7243
                     /vntifkey="51"
                     /label=DNA\insert
     CDS             4040..7234
                     /vntifkey="4"
                     /label=Expressed\protein
     misc_feature    4040..4087
                     /vntifkey="21"
                     /label=HA\signal\peptide
     misc_feature    4089..4117
                     /vntifkey="21"
                     /label=His10
     misc_feature    4127..4147
                     /vntifkey="21"
                     /label=TEV\protease\ENLYFQ*G
BASE COUNT     1847 a      2102 c      2021 g      1907 t
ORIGIN
        1 gacgcgccct gtagcggcgc attaagcgcg gcgggtgtgg tggttacgcg cagcgtgacc
       61 gctacacttg ccagcgccct agcgcccgct cctttcgctt tcttcccttc ctttctcgcc
```

121 acgttcgccg gctttccccg tcaagctcta aatcgggggc tccctttagg gttccgattt
181 agtgctttac ggcacctcga ccccaaaaaa cttgattagg gtgatggttc acgtagtggg
241 ccatcgccct gatagacggt ttttcgccct ttgacgttgg agtccacgtt ctttaatagt
301 ggactcttgt tccaaactgg aacaacactc aaccctatct cggtctattc ttttgattta
361 taagggattt tgccgatttc ggcctattgg ttaaaaaatg agctgattta acaaaaattt
421 aacgcgaatt ttaacaaaat attaacgttt acaatttcag gtggcacttt tcggggaaat
481 gtgcgcggaa cccctatttg tttatttttc taaatacatt caaatatgta tccgctcatg
541 agacaataac cctgataaat gcttcaataa tattgaaaaa ggaagagtat gagtattcaa
601 catttccgtg tcgcccttat tccctttttt gcggcatttt gccttcctgt ttttgctcac
661 ccagaaacgc tggtgaaagt aaaagatgct gaagatcagt tgggtgcacg agtgggttac
721 atcgaactgg atctcaacag cggtaagatc cttgagagtt ttcgccccga agaacgtttt
781 ccaatgatga gcacttttaa agttctgcta tgtggcgcgg tattatcccg tattgacgcc
841 gggcaagagc aactcggtcg ccgcatacac tattctcaga atgacttggt tgagtactca
901 ccagtcacag aaaagcatct tacggatggc atgacagtaa gagaattatg cagtgctgcc
961 ataaccatga gtgataacac tgcggccaac ttacttctga caacgatcgg aggaccgaag
1021 gagctaaccg cttttttgca caacatgggg gatcatgtaa ctcgccttga tcgttgggaa
1081 ccggagctga atgaagccat accaaacgac gagcgtgaca ccacgatgcc tgtagcaatg
1141 gcaacaacgt tgcgcaaact attaactggc gaactactta ctctagcttc ccggcaacaa
1201 ttaatagact ggatggaggc ggataaagtt gcaggaccac ttctgcgctc ggcccttccg
1261 gctggctggt ttattgctga taaatctgga gccggtgagc gtgggtctcg cggtatcatt
1321 gcagcactgg ggccagatgg taagccctcc cgtatcgtag ttatctacac gacggggagt
1381 caggcaacta tggatgaacg aaatagacag atcgctgaga taggtgcctc actgattaag
1441 cattggtaac tgtcagacca agtttactca tatatacttt agattgattt aaaacttcat
1501 ttttaattta aaaggatcta ggtgaagatc ctttttgata atctcatgac caaaatccct
1561 taacgtgagt tttcgttcca ctgagcgtca gaccccgtag aaaagatcaa aggatcttct
1621 tgagatcctt tttttctgcg cgtaatctgc tgcttgcaaa caaaaaaacc accgctacca
1681 gcggtggttt gtttgccgga tcaagagcta ccaactcttt ttccgaaggt aactggcttc
1741 agcagagcgc agataccaaa tactgtcctt ctagtgtagc cgtagttagg ccaccacttc
1801 aagaactctg tagcaccgcc tacatacctc gctctgctaa tcctgttacc agtggctgct
1861 gccagtggcg ataagtcgtg tcttaccggg ttggactcaa gacgatagtt accggataag
1921 gcgcagcggt cgggctgaac ggggggttcg tgcacacagc ccagcttgga gcgaacgacc
1981 tacaccgaac tgagatacct acagcgtgag cattgagaaa gcgccacgct tcccgaaggg
2041 agaaaggcgg acaggtatcc ggtaagcggc agggtcggaa caggagagcg cacgagggag
2101 cttccagggg gaaacgcctg gtatctttat agtcctgtcg ggtttcgcca cctctgactt
2161 gagcgtcgat ttttgtgatg ctcgtcaggg gggcggagcc tatggaaaaa cgccagcaac
2221 gcggcctttt tacggttcct ggccttttgc tggccttttg ctcacatgtt ctttcctgcg
2281 ttatcccctg attctgtgga taaccgtatt accgcctttg agtgagctga taccgctcgc
2341 cgcagccgaa cgaccgagcg cagcgagtca gtgagcgagg aagcggaaga gcgcctgatg
2401 cggtattttc tccttacgca tctgtgcggt atttcacacc gcatagacca gccgcgtaac
2461 ctggcaaaat cggttacggt tgagtaataa atggatgccc tgcgtaagcg ggtgtgggcg
2521 gacaataaag tcttaaactg aacaaaatag atctaaacta tgacaataaa gtcttaaact
2581 agacagaata gttgtaaact gaaatcagtc cagttatgct gtgaaaaagc atactggact
2641 tttgttatgg ctaaagcaaa ctcttcattt tctgaagtgc aaattgcccg tcgtattaaa
2701 gaggggcgtg gccaagggca tggtaaagac tatattcgcg gcgttgtgac aatttaccga
2761 acaactccgc ggccgggaag ccgatctcgg cttgaacgaa ttgttaggtg gcggtacttg
2821 ggtcgatatc aaagtgcatc acttcttccc gtatgcccaa ctttgtatag agagccactg
2881 cgggatcgtc accgtaatct gcttgcacgt agatcacata agcaccaagc gcgttggcct
2941 catgcttgag gagattgatg agcgcggtgg caatgccctg cctccggtgc tcgccggaga
3001 ctgcgagatc atagatatag atctcactac gcggctgctc aaacttgggc agaacgtaag
3061 ccgcgagagc gccaacaacc gcttcttggt cgaaggcagc aagcgcgatg aatgtcttac
3121 tacggagcaa gttcccgagg taatcggagt ccggctgatg ttgggagtag gtggctacgt
3181 ctccgaactc acgaccgaaa agatcaagag cagcccgcat ggatttgact tggtcagggc
3241 cgagcctaca tgtgcgaatg atgcccatac ttgagccacc taactttgtt ttagggcgac
3301 tgccctgctg cgtaacatcg ttgctgctgc gtaacatcgt tgctgctcca taacatcaaa
3361 catcgaccca cggcgtaacg cgcttgctgc ttggatgccc gaggcataga ctgtacaaaa
3421 aaacagtcat aacaagccat gaaaaccgcc actgcgccgt taccaccgct gcgttcggtc
3481 aaggttctgg accagttgcg tgagcgcata cgctacttgc attacagttt acgaaccgaa
3541 caggcttatg tcaactgggt tcgtgccttc atccgtttcc acggtgtgcg tcacccggca
3601 accttgggca gcagcgaagt cgaggcattt ctgtcctggc tggcgaacga gcgcaaggtt
3661 tcggtctcca cgcatcgtca ggcattggcg gccttgctgt tcttctacgg caaggtgctg
3721 tgcacggatc tgccctggct tcaggagatc ggaagacctc ggccgtcgcg gcgcttgccg
3781 gtggtgctga ccccggatga agtggttcgc atcctcggtt ttctggaagg cgagcatcgt
3841 ttgttcgccc aggactctag ctatagttct agtggttggc tacgtatact ccggaatatt
3901 aatagatcat ggagataatt aaaatgataa ccatctcgca aataaataag tattttactg
3961 ttttcgtaac agttttgtaa taaaaaaacc tataaatatt ccggattatt cataccgtcc
4021 caccatcggg cgcggatcc**a tgaaaactat tatcgccctg tcttacatct tctgtctcgt**
4081 **cttcgcacat caccaccacc accatcacca ccatcacggc gcgccggaaa acctgtactt**
4141 **ccagggtggc aacctcactg tggctgtggt cctgccactg gctaacactt cctacccttg**
4201 **gtcttgggct cgtgtgggtc cagctgtcga gctggccctg gctcaggtca aggccagacc**
4261 **tgacctgctg cccggctgga ccgtgcgcac tgtcctgggt tccagcgaga acgctctggg**
4321 **tgtgtgctct gacactgctg ctcctctggc tgctgtggac ctgaagtggg aacacaaccc**
4381 **agccgtgttc ctgggacctg gttgcgtgta cgctgccgct ccagtcggaa gattcaccgc**
4441 **tcactggcgc gtccctctgc tcactgctgg tgctcccgct ctgggattcg gagtgaagga**
4501 **cgagtacgct ctgaccactc gtgccggacc tagctacgct aagctgggtg acttcgtggc**
4561 **tgctctgcac cgccgtctgg gttgggagag gcaggccctg atgctgtacg cttacaggcc**
4621 **cggagacgag gaacactgct tcttcctggt ggaaggtctg ttcatgcgcg tccgtgacag**
4681 **gctgaacatc actgtggacc acctggagtt cgccgaagac gacctgtctc actacacccg**
4741 **cctgctgcgt actatgccca ggaagggcag agtgatctac atctgctctt caccagacgc**
4801 **tttccgcact ctcatgctcc tggctctgga ggctggcctg tgcggagaag actacgtctt**
4861 **cttccacctg gacatcttcg gtcagagcct gcagggaggt caaggaccag ctcctcgccg**
4921 **cccttgggag agaggtgacg gacaggacgt gtctgctcgc caggccttcc aggctgctaa**
4981 **gatcatcacc tacaaggacc ccgacaaccc agagtacctg gagttcctga agcagctgaa**
5041 **gcacctggcc tacgagcagt tcaacttcac tatggaggac ggcctggtca acactatccc**
5101 **cgctagcttc cacgacggac tgctgctgta catccaagct gtcactgaga ctctggctca**
5161 **cggcggaacc gtgactgacg gtgaaaacat cactcagagg atgtggaaca gatcattcca**
5221 **gggtgtgacc ggctacctga agatcgactc cagcggcgac cgtgagactg acttctccct**
5281 **gtgggacatg gacccagaaa acggcgcctt cagggtggtc ctgaactaca acggaaccag**
5341 **ccaggagctg gtggctgtct ctggaaggaa gctgaactgg ccactgggtt accctccccc**

5401 **agacatccct aagtgcggtt tcgacaacga ggaccccgcc tgcaaccagg accacctgtc**

5461 **cactctggaa gtgctggctc tggtcggatc tctgtcactg ctgggtatcc tgatcgtctc**

5521 **attcttcatc taccgcaaga tgcagctgga gaaggaactg gcctccgagc tgtggagagt**

5581 **gcgctgggag gacgtcgaac catcttcact ggaacgccac ctgcgttccg ctggaagccg**

5641 **tctgaccctg tctggaaggg gttcaaacta cggctccctg ctgaccactg agggacagtt**

5701 **ccaggtgttc gctaagactg cctactacaa gggtaacctg gtggccgtca agcgtgtcaa**

5761 **ccgtaagagg atcgagctga ccaggaaggt gctgttcgaa ctgaagcaca tgcgtgacgt**

5821 **ccagaacgag cacctgacca ggttcgtggg cgcttgcact gaccctccca acatctgcat**

5881 **cctgactgaa tactgcccta gaggatcact gcaggacatc ctggagaacg aatccatcac**

5941 **cctggactgg atgttccgct acagcctgac taacgacatc gtgaagggca tgctgttcct**

6001 **gcacaacggc gctatctgct ctcacggaaa cctgaagtcc agcaactgcg tggtggacgg**

6061 **tagattcgtc ctgaagatca ccgactacgg cctggagtca ttccgcgacc tggaccctga**

6121 **acagggccac accgtgtacg ccaagaagct gtggactgct cccgaactgc tgagaatggc**

6181 **tagcccacct gtgcgcggct ctcaggctgg agacgtctac tccttcggaa tcatcctgca**

6241 **ggagatcgct ctgcgtagcg gtgtgttcca cgtcgaaggc ctggacctgt ctccaaagga**

6301 **gatcatcgaa agggtgacca gaggagagca gcccccattc aggccttcac tggccctgca**

6361 **gtcccacctg gaggaactgg gtctgctgat gcagagatgc tgggctgagg accctcagga**

6421 **acgccctccc ttccagcaga tccgcctgac tctgcgtaag ttcaacaggg agaactcttc**

6481 **aaacatcctg gacaacctgc tgagcagaat ggaacagtac gccaacaacc tggaggaact**

6541 **ggtcgaggaa cgtacccagg cttacctgga ggaaaagagg aaggctgagg ccctgctgta**

6601 **ccagatcctg ccccactctg tggctgaaca gctgaagcgc ggtgaaaccg tgcaggctga**

6661 **agccttcgac tcagtcacta tctacttctc cgacatcgtg ggattcaccg ctctgtctgc**

6721 **cgagtcaacc ccaatgcagg tggtcactct gctgaacgac ctgtacactt gcttcgacgc**

6781 **cgtcatcgac aacttcgacg tgtacaaggt cgaaaccatc ggtgacgctt acatggtggt**

6841 **gtccggactg ccagtccgca acggtcgtct gcacgcttgc gaagtggcca gaatggctct**

6901 **ggctctgctg gacgccgtgc gctccttcag aatccgccac cgtcctcagg agcagctgag**

6961 **gctgagaatc ggtatccaca ctggcccagt ctgcgctgga gtggtcggtc tgaagatgcc**

7021 **tcgttactgc ctgttcggtg acaccgtgaa cactgccagc aggatggagt ctaacggcga**

7081 **agctctgaag atccacctgt ccagcgaaac caaggctgtc ctggaggagt tcggtggctt**

7141 **cgagctggaa ctgcgcggcg acgtggaaat gaagggaaaa ggcaaagtca ggacatactg**

7201 **gttgttggga gaaagaggta gcagcacccg tgga**taaaag cttgtcgaga agtactagag

7261 gatcataatc agccatacca catttgtaga ggttttactt gctttaaaaa acctcccaca

7321 cctccccctg aacctgaaac ataaaatgaa tgcaattgtt gttgttaact tgtttattgc

7381 agcttataat ggttacaaat aaagcaatag catcacaaat ttcacaaata aagcattttt

7441 ttcactgcat tctagttgtg gtttgtccaa actcatcaat gtatcttatc atgtctggat

7501 ctgatcactg cttgagccta ggagatccga accagataag tgaaatctag ttccaaacta

7561 ttttgtcatt tttaattttc gtattagctt acgacgctac acccagttcc catctatttt

7621 gtcactcttc cctaaataat ccttaaaaac tccatttcca cccctcccag ttcccaacta

7681 ttttgtccgc ccacagcggg gcatttttct tcctgttatg tttttaatca aacatcctgc

7741 caactccatg tgacaaaccg tcatcttcgg ctacttttc tctgtcacag aatgaaaatt

7801 tttctgtcat ctcttcgtta ttaatgtttg taattgactg aatatcaacg cttatttgca

7861 gcctgaatgg cgaatgg

//

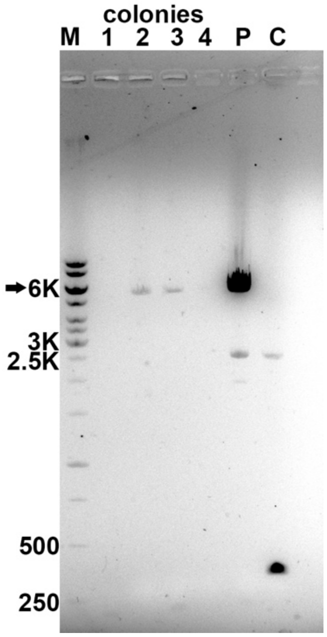

Supplementary Figure S3. PCR verification of recombinant bacmid production in four isolated white colonies and one amplified purified bacmid DNA derived from the selected white colony. The expected size of the bacmid containing the full-length pGC-A is 5,495 bp. Empty bacmid yields 300 bp. Bacmid containing pFastBac1 empty vector is 2,300 bp. Abbreviations: M, molecular weight marker; colonies 1 to 4, colony PCR using individual white colonies; P, PCR using purified bacmid DNA; C, blue colony negative control (empty bacmid).

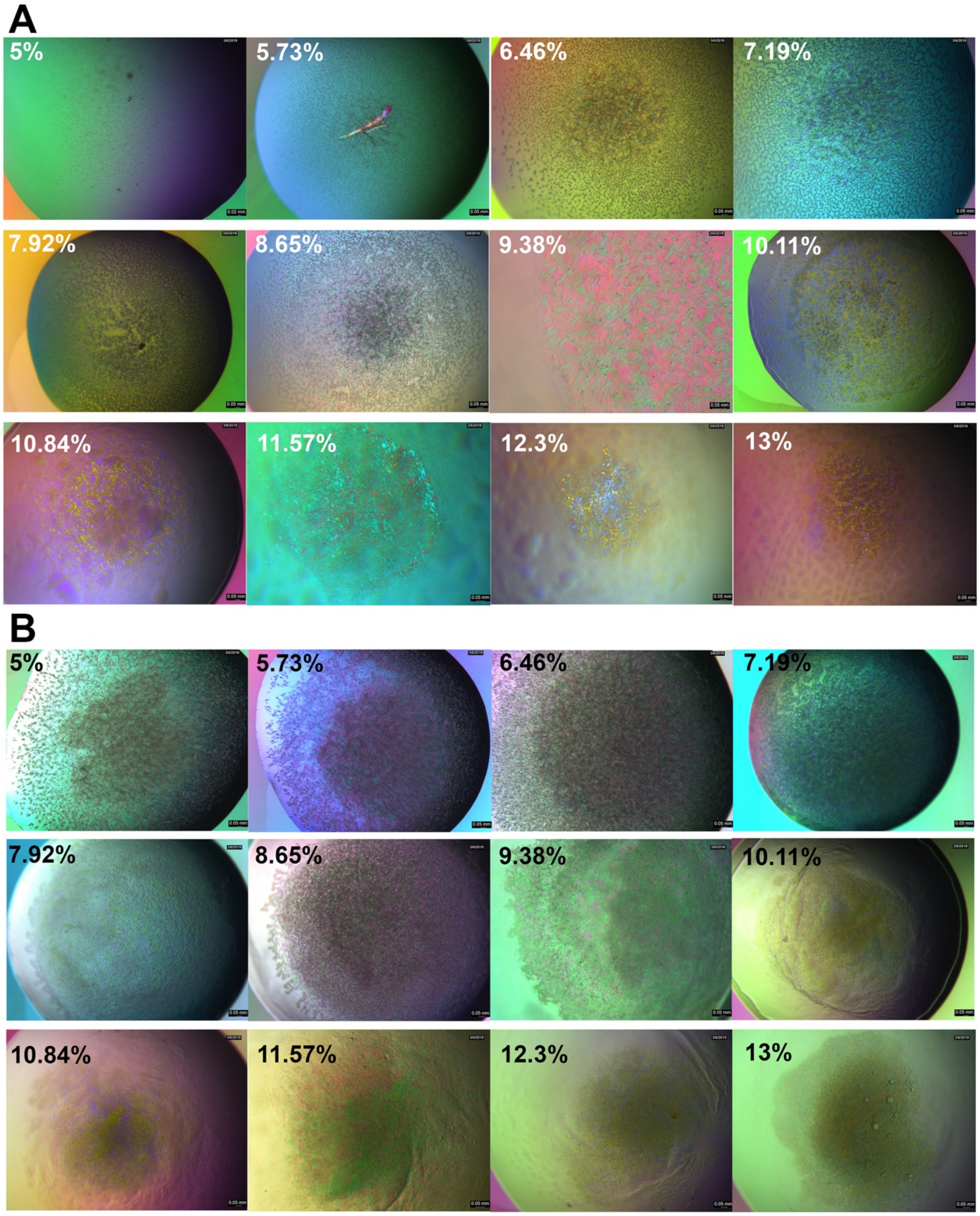

Supplementary Figure S4. Summary of PEG 3350 screening crystallization condition with two protein concentrations. Shown are visible light images of crystallization drops in 24-well plates. (**A**) Screening in 5–13% PEG 3350 at a protein concentration of 1.18 mg/mL.
(**B**) Screening in 5–13% PEG 3350 at a protein concentration of 2.25 mg/mL.

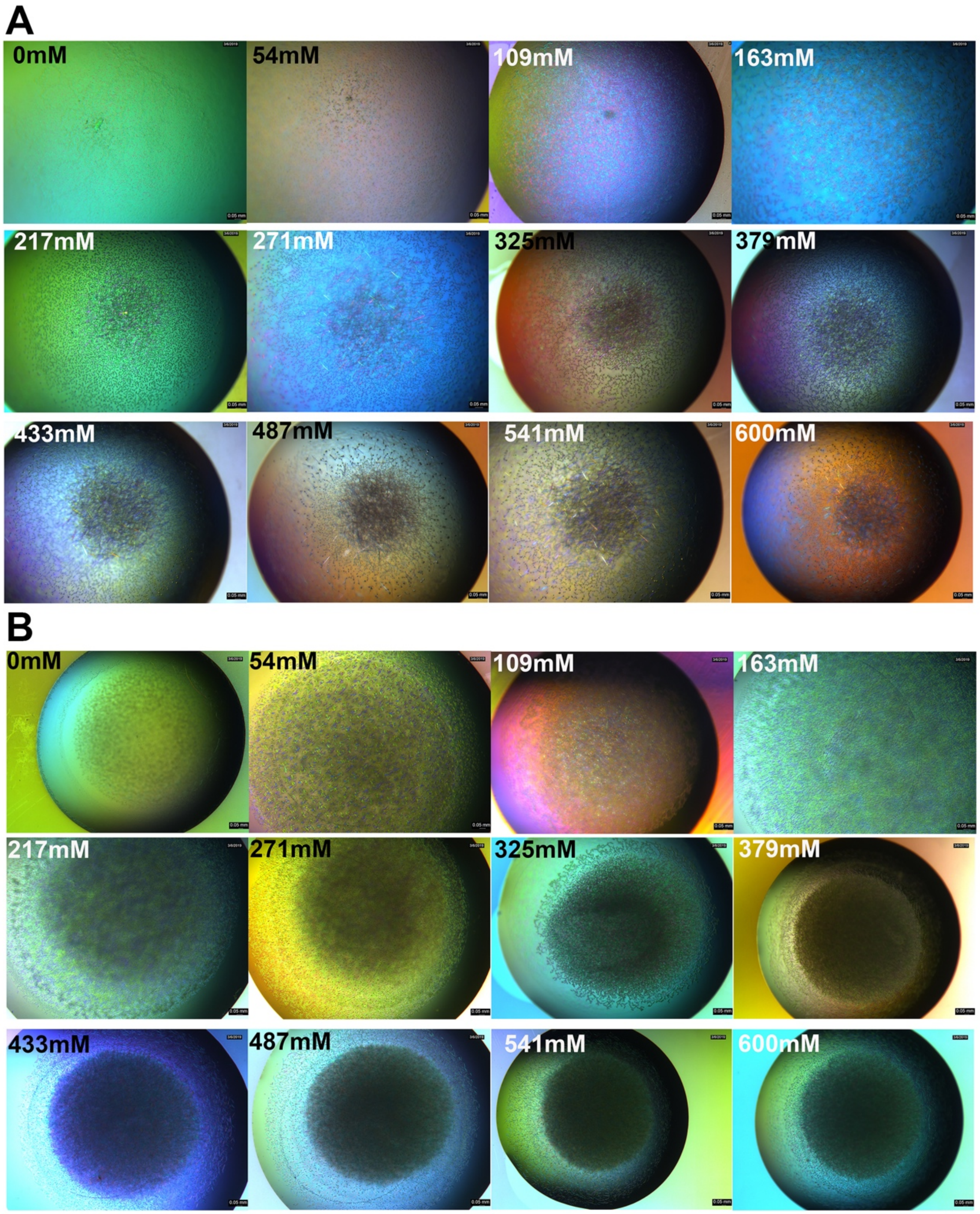

Supplementary Figure S5. Summary of ammonium sulfate screening with two protein concentrations. Shown are visible light images of crystallization drops in 24-well plates. (**A**) Screening in 0–0.6 M ammonium sulfate at a protein concentration of 1.18 mg/mL. (**B**) Screening in 0–0.6 M ammonium sulfate at a protein concentration of 2.25 mg/mL.

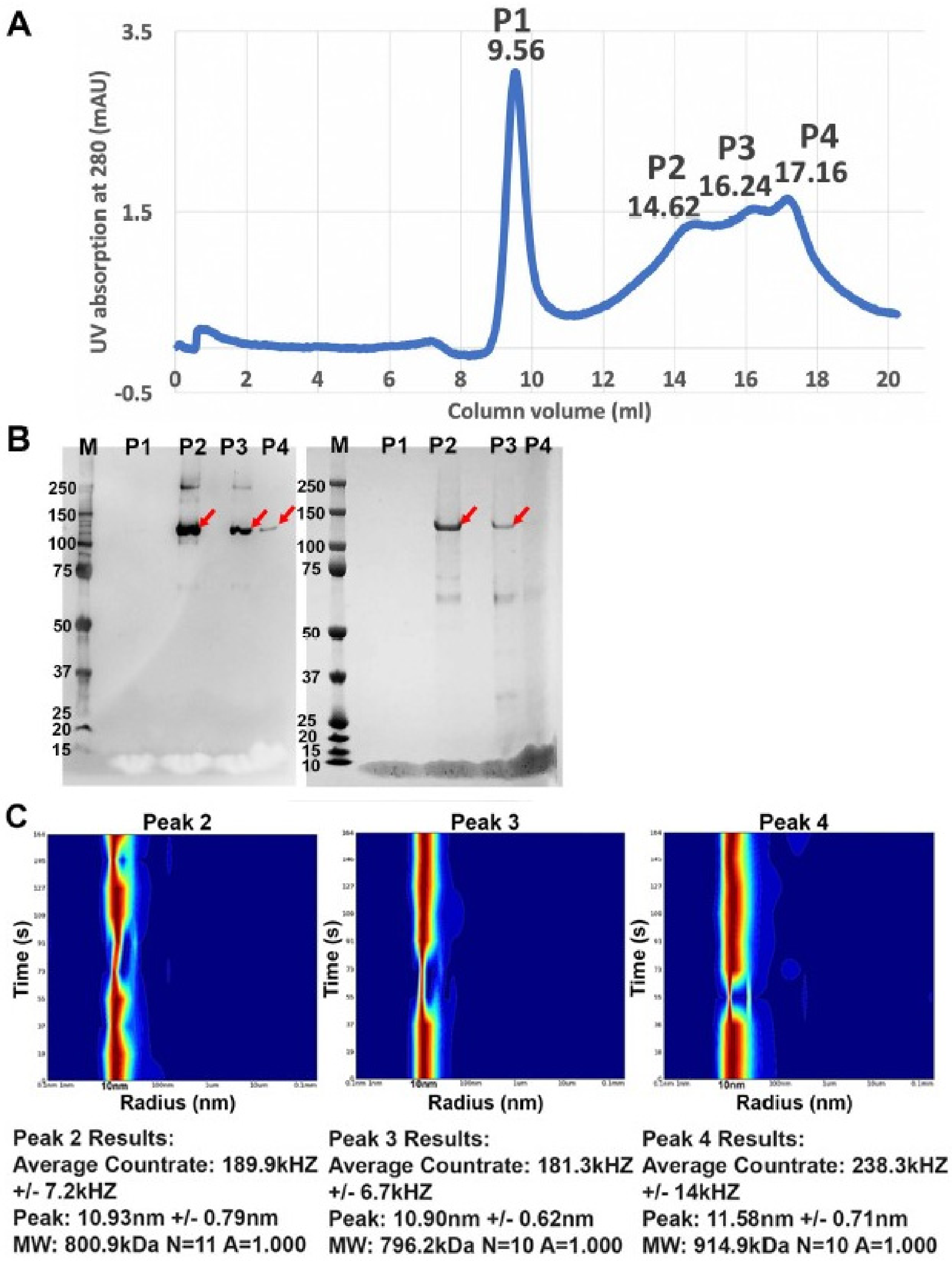

Supplementary Figure S6. Summary of characterization of different oligomeric peak fractions from Superose6 column. (**A**) Shown in the elution profile are Peaks P2, P3 and P4, which are consistent with tetramer, dimer and monomer, respectively. Peak P1 is in the void volume. (**B**) Western blotting (left; anti-pGC-A) and Coomassie Blue staining (right) of Peaks P1-P4. Peak P1 lacked visible pGC-A by either method. pGC-A (red arrow) is ~120 kDa. (**C**) Dynamic light scattering (performed as described previously[63]) of concentrated protein samples from Peaks P2, P3, and P4

Figure 1

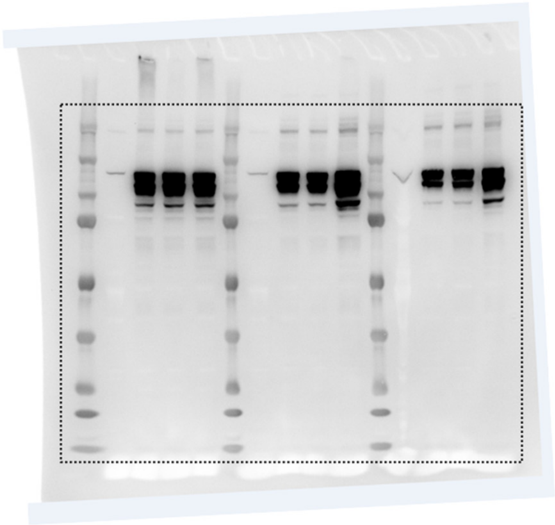

Figure 3A

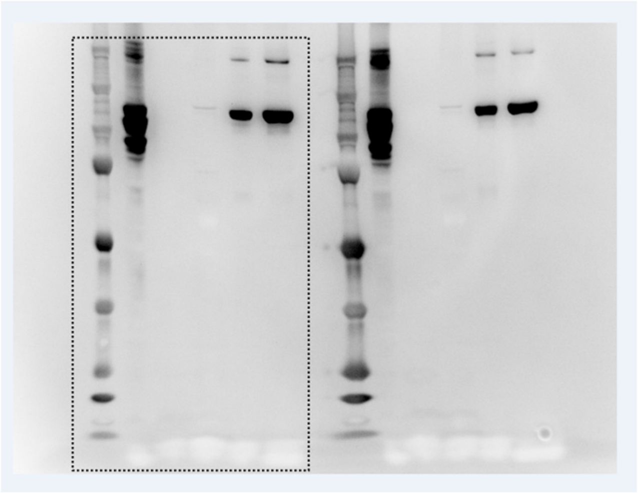

Figure 3B

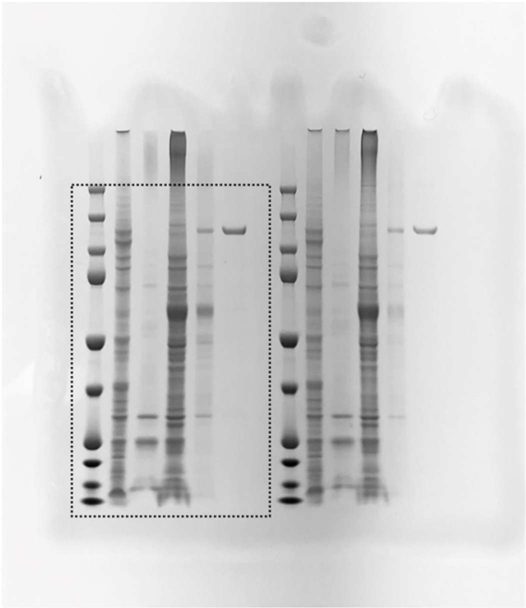

Figure 3C

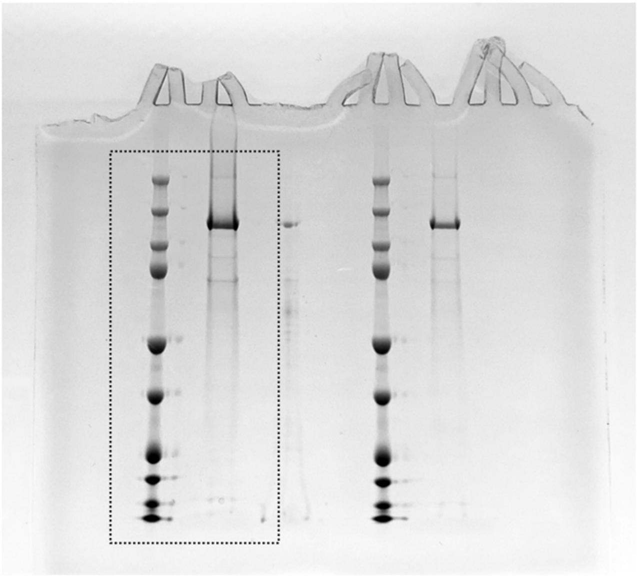

Supplementary Figure S7. Uncropped images from all gels and immunoblots.

Figure 5

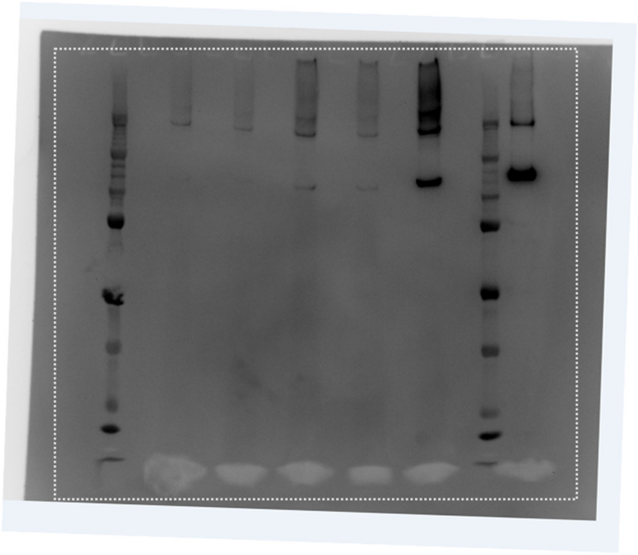

Figure 8

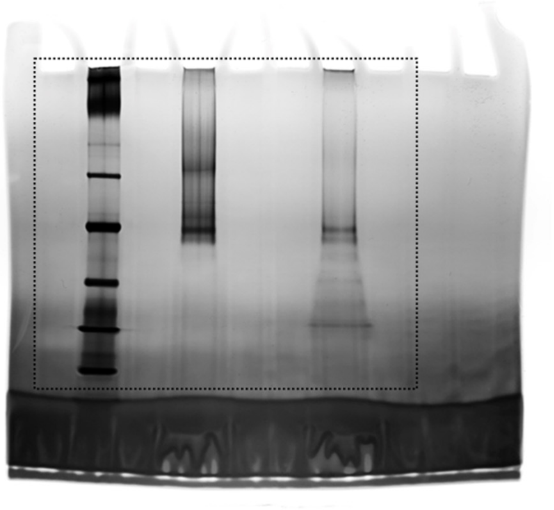

Figure 9

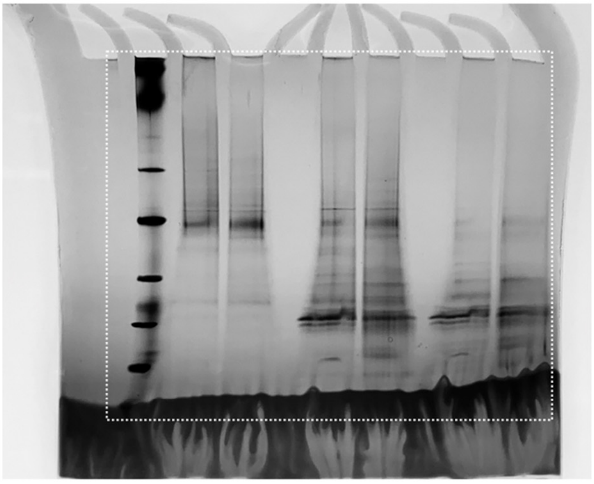

Figure S3

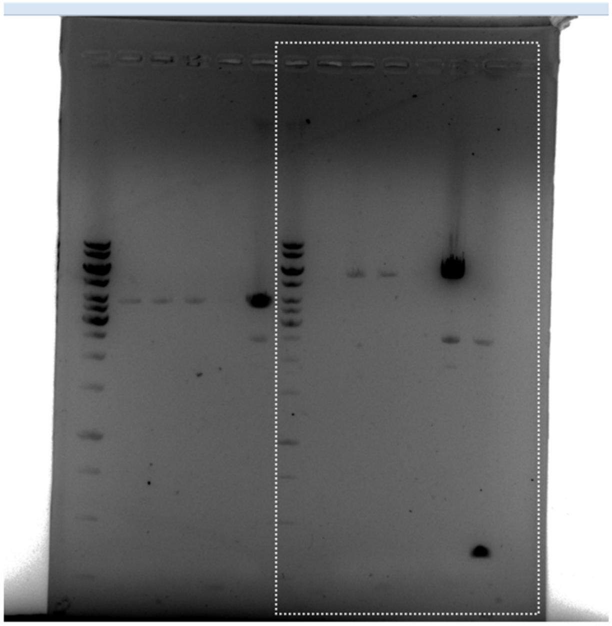

Supplementary Figure S7 (continued). Uncropped images from all gels and immunoblots.

Supplementary References

60. Janakiraman, V., Forrest, W. F., Chow, B. & Seshagiri, S. A rapid method for estimation of baculovirus titer based on viable cell size. *J Virol Methods* **132**, 48-58 (2006). http://dx.doi.org/10.1016/j.jviromet.2005.08.021
61. Janakiraman, V., Forrest, W. F. & Seshagiri, S. Estimation of baculovirus titer based on viable cell size. *Nat Protoc* **1**, 2271-2276 (2006). http://dx.doi.org/10.1038/nprot.2006.387
62. Wittig, I., Karas, M. & Schagger, H. High resolution clear native electrophoresis for in-gel functional assays and fluorescence studies of membrane protein complexes. *Mol Cell Proteomics* **6**, 1215-1225 (2007). http://dx.doi.org/10.1074/mcp.M700076-MCP200
63. Nagaratnam, N. *et al.* Structural insights into the function of the catalytically active human Taspase1. *Structure* **29**, 873-885 e875 (2021). http://dx.doi.org/10.1016/j.str.2021.03.008
